# *Lnk* deficiency enhances translesion synthesis to alleviate replication stress and promote hematopoietic stem cell fitness

**DOI:** 10.1172/JCI191713

**Published:** 2025-10-30

**Authors:** Brijendra Singh, Md Akram Hossain, Xiao Hua Liang, Jeremie Fages, Carlo Salas Salinas, Roger A. Greenberg, Wei Tong

**Affiliations:** 1 Division of Hematology, Children’s Hospital of Philadelphia, Philadelphia, Pennsylvania, USA.; 2 Division of Pediatrics and; 3 Division of Cancer Biology, Perelman School of Medicine at the University of Pennsylvania, Philadelphia, Pennsylvania, USA.

**Keywords:** Development, Hematology, Cell stress, DNA repair, Hematopoietic stem cells

## Abstract

The adaptor protein LNK/SH2B3 negatively regulates hematopoietic stem cell (HSC) homeostasis. *Lnk-*deficient mice show marked expansion of HSCs without premature exhaustion. *Lnk* deficiency largely restores HSC function in Fanconi anemia (FA) mouse models and primary FA patient cells, albeit protective mechanisms remain enigmatic. Here, we uncover a role for LNK in regulating translesion synthesis (TLS) during HSC replication. *Lnk* deficiency reduced replication stress–associated DNA damage, particularly in the FA background. *Lnk* deficiency suppressed single-strand DNA breaks, while enhancing replication fork restart in FA-deficient HSCs. Diminished replication-associated damage in *Lnk*-deficient HSCs occurred commensurate with reduced ATR/p53 checkpoint activation that is linked to HSC attrition. Notably, *Lnk* deficiency ameliorated HSC attrition in FA mice without exacerbating carcinogenesis during aging. Moreover, we demonstrated that enhanced HSC fitness from *Lnk* deficiency was associated with increased TLS activity via REV1 and, to a lesser extent, TLS polymerase eta (η). TLS polymerases are specialized to execute DNA replication in the presence of lesions or natural replication fork barriers that stall replicative polymerases. Our findings implicate elevated use of these specialized DNA polymerases as critical to the enhanced HSC function imparted by *Lnk* deficiency, which has important ramifications for stem cell therapy and regenerative medicine in general.

## Abstract 2

Deleting an adaptor protein boosts the ability of blood stem cells to regenerate with less DNA damage and may offer new treatments for Fanconi Anemia.

## Graphical abstract

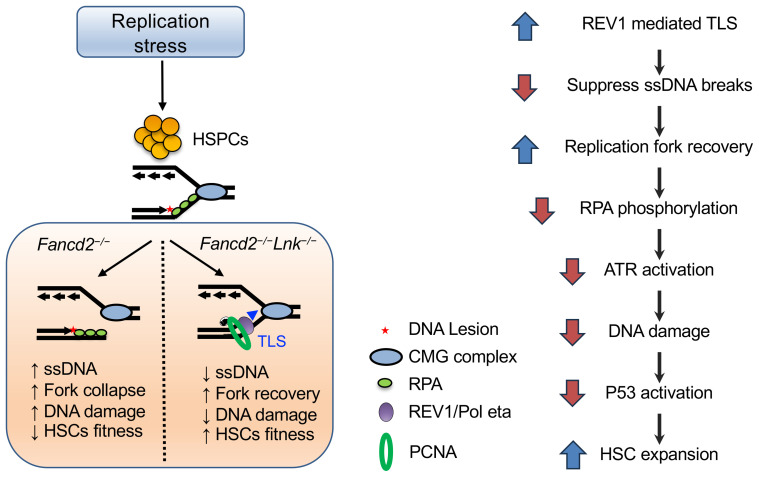

## Introduction

Hematopoietic stem cells (HSCs) are endowed with the ability to self-renew and differentiate, thus sustaining a lifelong supply of blood cells. Disruption of HSC homeostasis is associated with a variety of human disorders (1, 2). Faithful maintenance of genome integrity in hematopoietic stem and progenitor cells (HSPCs) is crucial to hematopoiesis and suppression of blood cancers. One of the most common inherited bone marrow failure syndromes, Fanconi anemia (FA), is caused by mutations in one of 23 FA complement genes. A network of FA proteins cooperates to repair DNA interstrand cross-link damage (3) and relieve replication stress (4), resulting in progressive HSPC decline and increased leukemia/cancer incidence (5, 6).

The adaptor protein LNK (SH2B3) is a negative regulator of HSC homeostasis (7, 8). *Lnk^–/–^* mice exhibit elevated blood counts (9), bone marrow (BM) progenitors (10), and a remarkable more-than-10-fold increase in HSC numbers with superior self-renewal ability (7, 11, 12). *Lnk^–/–^* mice are unique in harboring a markedly expanded HSC pool with multilineage reconstitution and serial transplantability without premature exhaustion (13). We previously reported that loss of *Lnk* partially restores HSC function in both FA mouse models and human HSPCs from FA patients (14, 15). Importantly, LNK does not directly participate in interstrand cross-link DNA repair; rather, its loss ameliorates replication stress (14). The mechanisms by which *Lnk* deficiency reduces FA severity and promotes HSC fitness remain to be established.

DNA replication stress, defined as the slowing or stalling of replication forks, is considered an emerging hallmark of cancer and a major contributor to genomic instability (16). It is also prevalent in early mammalian development, resulting in genome instability and aneuploidy, constituting a barrier to development (17). Importantly, replication stress is a potent driver of stem cell functional decline (18). Besides exogenous DNA-damaging agents, endogenous sources of replication stress arise from spontaneous DNA lesions, nucleotide imbalance/depletion, RNA-DNA hybrids, transcription-replication conflicts, or difficult-to-replicate genomic features, including fragile sites, repetitive DNA, and secondary DNA structures (19).

DNA damage tolerance (DDT) mechanisms play a key role in maintaining genome stability against exogenous and endogenous stress. DDT utilizes specialized DNA polymerases to bypass DNA lesions, allowing complete replication without replication fork collapse, thus preventing severe DNA damage (20). One such DDT mechanism is translesion synthesis (TLS), where replicative DNA polymerase is temporarily replaced by one of the TLS polymerases that can replicate across DNA lesions. The human genome encodes 17 DNA polymerases (Pols) (21). While Pols alpha (α), delta (δ), epsilon (ε), and gamma (γ) are responsible for replicating the bulk of nuclear and mitochondrial DNA, the rest specialize in translesion and repair synthesis. TLS is a mechanism whereby specialized DNA polymerases tolerate various DNA lesions to allow for the continuation of DNA replication (22). Even though TLS polymerases have higher intrinsic error rates than replicative polymerases, they increase genome stability and reduce tumorigenesis ostensibly because they allow replication to proceed across blocking lesions that otherwise create replication fork collapse and chromosome breaks, which bear far worse consequences (21). TLS polymerases are best characterized by the Y family, encompassing Pols kappa (κ), eta (η), iota (ι), and REV1. TLS is mediated by 2 overlapping but distinct pathways, K^164^-monoubiquitination of proliferating cell nuclear antigen (PCNA; a DNA sliding clamp and DNA polymerase processivity factor) and REV1, to recruit TLS polymerases to replication forks (20). TLS polymerases insert bases opposite the lesion as an “inserter.” Extension from the incorporated base is largely carried out by B-family polymerase Pol zeta (ζ) complex via REV1 (19). *Rev1^–/–^* and *Pcna^K164R^* single-mutant mice are grossly normal but show compromised HSPC functions (23, 24). *Rev1^–/–^* *Pcna^K164R^* double-mutant mice are embryonic lethal from hematopoietic failure, indicating a critical role for TLS in hematopoiesis (25).

While best studied for their ability to bypass physical lesions on the DNA, accumulating evidence suggests a non-canonical role for TLS polymerases in coping with various natural replication fork barriers and alleviating replication stress (21, 26, 27). One notable example is Polη, which replicates efficiently and accurately past ultraviolet (UV) pyrimidine dimers. Mutations in the *POLH* gene that encode Polη are responsible for the variant form of xeroderma pigmentosum (XP-V), a rare autosomal recessive disorder characterized by extreme sensitivity to sunlight and a very high incidence of skin cancer (28). Importantly, recent evidence implicates a role for Polη in maintaining chromosomal stability and preventing common fragile site breakage during the unperturbed S phase, irrespective of UV damage, implicating it in the resolution of other stresses (29–31). The non-canonical activities of Polη derive from its enzymatic capacity to accommodate a variety of altered DNA structures, including sequences that form hairpins or G-quadruplexes at common fragile sites or telomeric DNA structures (32). These DNA replication impediments would otherwise block replicative DNA Pols (δ and ε). Thus, Polη mitigates replication stress and promotes genome stability in S-phase cells by carrying out DNA synthesis at difficult-to-replicate regions of the genome that form natural replication fork barriers (32).

In this study, we performed a comprehensive investigation on how *Lnk* deficiency ameliorates HSC defects associated with FA and how *Lnk* deficiency increases HSC fitness in general. Our work uncovered a role for the LNK-regulated TLS polymerases in controlling HSC expansion upon replication stress. Our data suggest that the loss of *Lnk* promotes TLS activities to facilitate DNA replication and reduce replication-associated DNA damage, thus reducing ATR/p53 checkpoint activation and HSC attrition. The mechanisms by which *Lnk* inhibition alleviates endogenous replication stress and promotes HSC fitness have important ramifications for stem cell therapy and regenerative medicine in general.

## Results

### Lnk deficiency reduces DNA damage in Fancd2^–/–^ HSPCs at the steady state and upon replication stress.

HSPC decline in FA patients is attributed to elevated DNA damage. To explore the mechanisms by which *Lnk* deficiency ameliorates HSPC defects associated with FA, we set out to examine DNA damage at the steady state and upon replication stress under various conditions.

**Figure 1:**
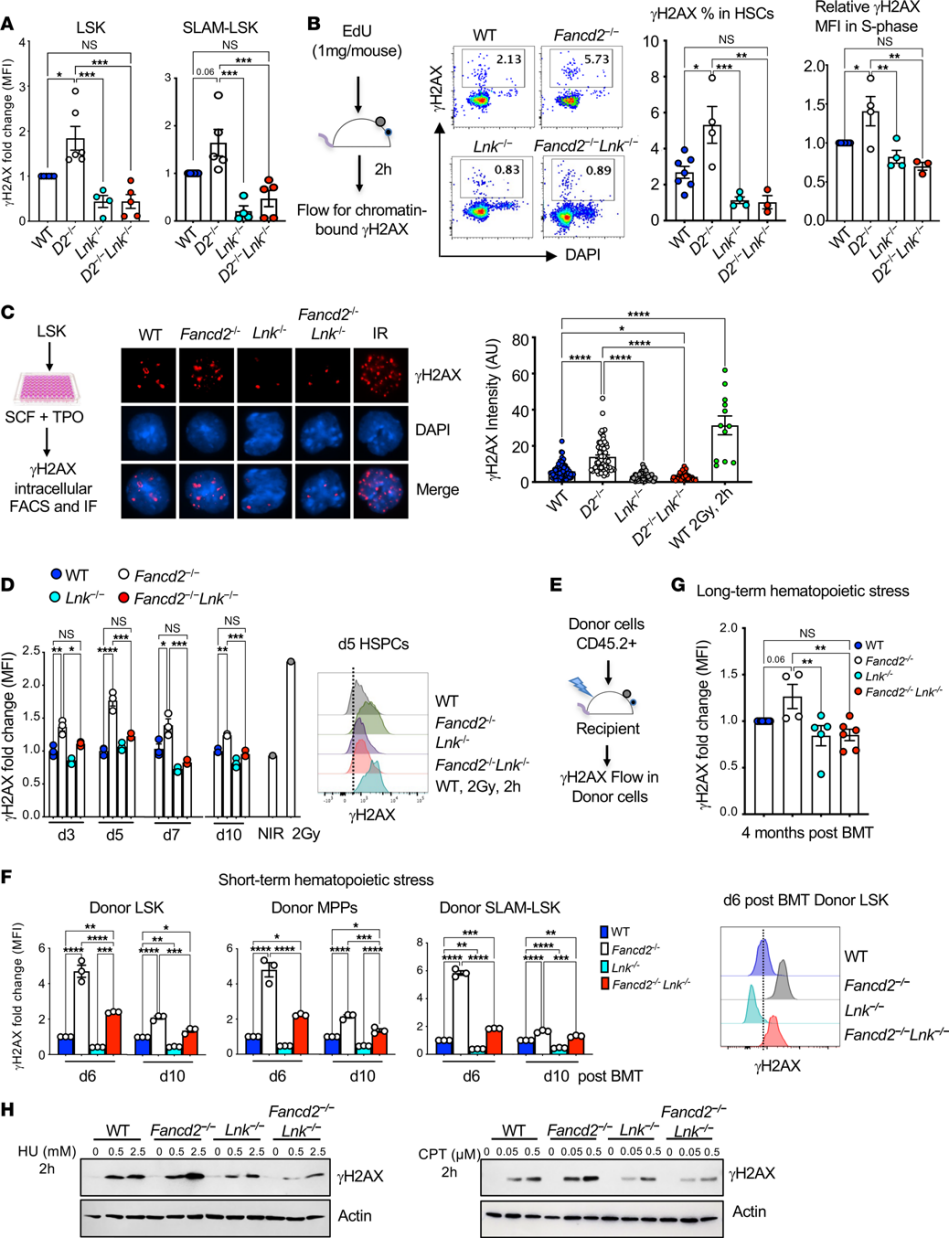
*Lnk* deficiency reduces DNA damage in *Fancd2^–/–^* HSPCs at steady state and upon replication stress. (**A** and **B**) Comparison of levels of DNA damage in HSCs and HSPCs at steady state. (**A**) Quantification of γH2AX (fold change in MFI as normalized to WT) within LSK and SLAM LSK populations analyzed by flow cytometry. (**B**) Experimental design, representative flow cytometry plots, and quantification of chromatin-bound γH2AX within the HSC population. EdU was injected 2 hours before BM harvest, and percentage γH2AX^+^ cells and fold change in MFI of γH2AX within the S phase of HSCs as determined by EdU^+^ cells are shown. (**C**) Comparison of levels of DNA damage in HSPCs in ex vivo culture. Experimental design for ex vivo LSK culture, representative immunofluorescence images of γH2AX foci, and quantification of fluorescence intensity in day 3 HSPCs are shown. IR, irradiated. Original magnification, ×63. (**D**) LSK cells were cultured, and γH2AX levels were examined by flow cytometry at the indicated days. Left: Quantification of fold change in MFI of γH2AX in ex vivo–cultured HSPCs. Right: Representative histogram plots for γH2AX in day 5 HSPCs. NIR, non-irradiated. WT cells 2 hours after 2 Gy x-ray irradiation were used as a positive control. (**E**–**G**) Comparison of levels of DNA damage in HSCs and HSPCs upon replication stress in vivo. (**E**) Experimental design for γH2AX examination within donor cells of LSK during transplantation-induced replication stress. (**F**) Quantification of fold changes in MFI of γH2AX within donor LSK cells, MPPs, and HSCs (SLAM LSK), days 6 and 10 after BMT (short-term hematopoietic stress), and a representative histogram plot for γH2AX within donor LSK cells, day 6 after BMT. (**G**) Fold changes in MFI of γH2AX within donor LSK cells 16 weeks after BMT (long-term hematopoietic stress). (**H**) Immunoblots showing the γH2AX level in freshly sorted LK cells treated with HU or CPT for 2 hours. In all relevant panels, each symbol represents an individual mouse; bars indicate mean values; error bars indicate SEM. *P* values were calculated using 1-way ANOVA; *P < 0.05, **P < 0.01, ***P < 0.001, ****P < 0.0001.

We first measured the phosphorylation of H2AX (pSer139), or γH2AX, a common DNA damage marker, in both HSPC (LSK) and HSC (SLAM LSK) populations at the steady state using fluorescence-activated cell sorting (FACS). *Fancd2^–/–^* HSPCs indeed had significantly increased γH2AX as compared with wild-type (WT) controls, while *Lnk^–/–^* HSPCs showed significantly lower γH2AX levels than those of *Fancd2^–/–^*. Notably, loss of *Lnk* significantly reduced the γH2AX level in *Fancd2^–/–^* HSPCs (Figure 1A). To precisely assess γH2AX on the DNA, we developed a FACS strategy to quantify chromatin-bound γH2AX in HSCs in different cell cycle stages in vivo (Figure 1B). We injected EdU (5-ethynyl-2′-deoxyuridine) into mice 2 hours before BM harvest. BM cells were subjected to Triton-based pre-extraction to remove soluble γH2AX before fixation. The cells were stained with antibodies against HSC surface markers and γH2AX followed by Click chemistry–based EdU staining for the cell cycle. *Fancd2^–/–^* HSCs showed increased percentage γH2AX^+^ cells and chromatin-bound γH2AX levels in the S phase compared with WT, while *Lnk* deficiency significantly reduced them (Figure 1B). Proliferation-associated stress is a major cause of DNA damage. To test whether deficiency of *Lnk* reduces replication-associated DNA damage in ex vivo culture, we purified LSK cells and cultured them in medium containing a cocktail of cytokines, then examined γH2AX^+^ nuclear foci by immunofluorescence or FACS. A higher mean fluorescence intensity of γH2AX foci was observed in *Fancd2^–/–^* HSPCs, with the majority of the cells having more than four γH2AX foci, indicative of prolonged DNA damage in *Fancd2^–/–^* HSPCs. Importantly, *Lnk^–/–^* and *Fancd2^–/–^ Lnk^–/–^* HSPCs had reduced γH2AX foci (Figure 1C). We also measured the γH2AX levels at different time intervals using FACS. HSPCs exposed to x-ray irradiation were analyzed in parallel as a positive control. *Fancd2^–/–^* HSPCs had significantly increased γH2AX levels that peaked at day 5. Importantly, the loss of *Lnk* significantly reduced the γH2AX levels at all time points (Figure 1D). The majority of HSCs are quiescent at the steady state. However, in response to stress signals such as transplantation into lethally irradiated animals, HSCs exit quiescence and undergo extensive proliferation and differentiation. To gain insights into replication-associated DNA damage in vivo, we measured DNA damage of the donor cells in the short term and long term after BM transplantation (BMT) (Figure 1E). At days 6 and 10 of short-term BMT, donor *Fancd2^–/–^* cells showed significantly increased γH2AX levels compared with those of WT. Notably, loss of *Lnk* significantly reduced the γH2AX levels in *Fancd2^–/–^* HSPCs (Figure 1F). Similar results were obtained during long-term replication stress 4 months after BMT (Figure 1G). Furthermore, we examined DNA damage in response to exogenous replication stressors, hydroxyurea (HU), which depletes dNTPs, and camptothecin (CPT), which inhibits topoisomerase I. Freshly sorted LK (lineage^–^c-Kit^+^) cells were treated with a graded dose of HU or CPT for 2 hours, and then the cell lysates were subjected to Western blotting (WB) for γH2AX. Both drugs increased γH2AX levels in a dose-dependent manner. The γH2AX level was more pronounced in *Fancd2^–/–^* cells, while *Lnk* deficiency reduced it (Figure 1H). Together, these data suggest that *Lnk* deficiency reduced replication-associated DNA damage in *Fancd2^–/–^* HSPCs during normal hematopoiesis and upon replication stress.

### Lnk deficiency moderately promotes survival of Fancd2^–/–^ mice upon chronic replication stress, and Lnk deficiency does not synergize with FA to exacerbate malignancies during aging.

**Figure 2:**
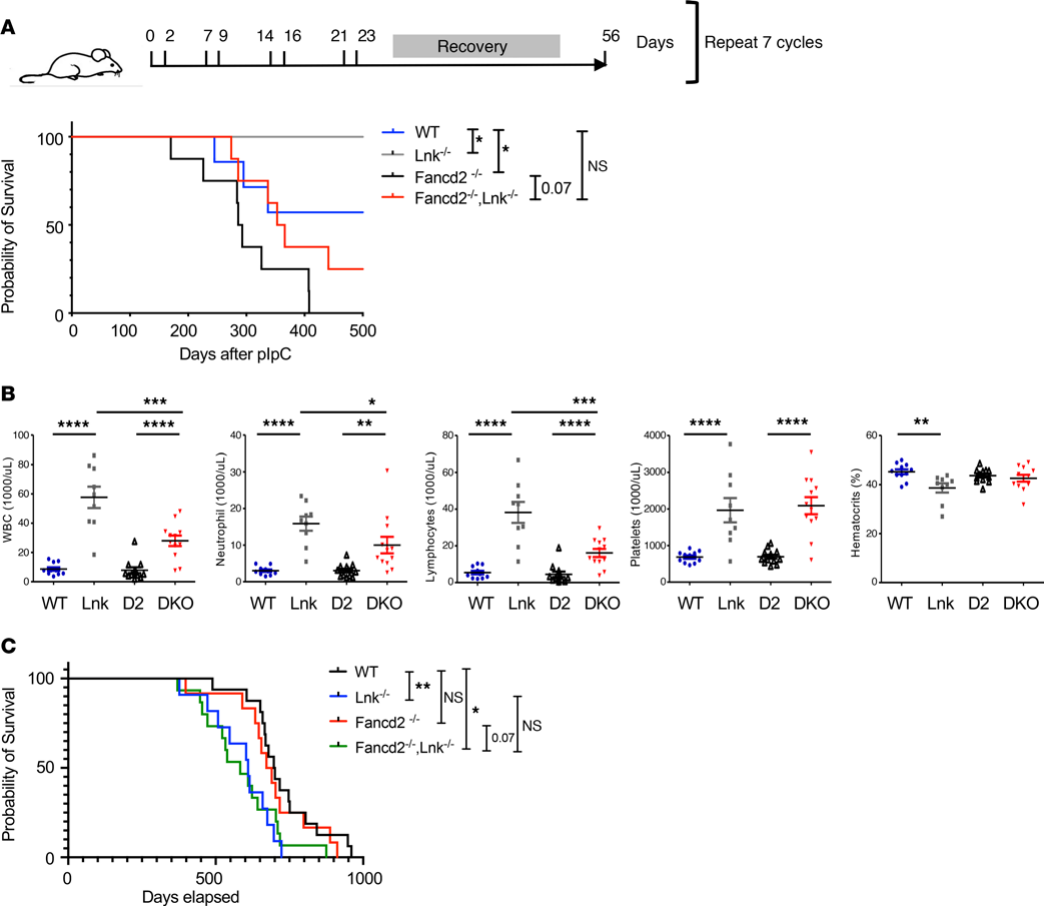
*Lnk* deficiency moderately promotes survival of *Fancd2^–/–^* mice upon chronic replication stress, and combined loss of *Lnk* and *Fancd2* does not exacerbate malignancies during aging. (**A**) The top diagram depicts a schematic overview of chronic stress induced by repeated pIpC injection. Mice were injected i.p. with pIpC twice per week for 4 weeks, followed by 4 weeks of rest. This 8-week cycle was repeated 7 times. Event-free survival is graphed in Kaplan-Meier curves. *n* = 7–9 mice per group. *P* values are calculated by log-rank analysis. *P < 0.05. (**B**) Complete blood count analysis of peripheral blood from WT, *Fancd2^–/–^* (D2), *Lnk^–/–^* (Lnk), and *Fancd2^–/–^ Lnk^–/–^* (DKO) mice, aged between 12 and 18 months. WBC, white blood cell. Each symbol represents an individual mouse. Horizontal lines indicate mean frequencies, and error bars indicate SEM. *P* values were calculated using 1-way ANOVA; *P < 0.05, **P < 0.01, ***P < 0.001, ****P < 0.0001. (**C**) A cohort of mice was observed for aging analysis. *n* = 11–16 per group. Event-free survival is graphed in Kaplan-Meier curves. *P* values were calculated by log-rank analysis. *P < 0.05, **P < 0.01.

Since *Lnk* deficiency reduces DNA damage upon short-term replication stress, we next asked whether it would also increase resistance to chronic replication stress in vivo. Published work from the Milsom laboratory showed that prolonged forced proliferation by repeated polyinosinic-polycytidylic acid (pIpC) injection induces pancytopenia in *Fanca*-deficient mice (33). We thus subjected WT, *Fancd2^–/–^*, *Lnk^–/–^*, and *Fancd2^–/–^ Lnk^–/–^* mice to an extended pIpC regimen, i.e., pIpC injection 2 times per week for 4 weeks followed by 4 weeks of rest. We repeated this treatment for 7 cycles and found that *Lnk^–/–^* mice survived significantly longer than WT mice, while *Fancd2^–/–^* mice died faster (Figure 2A). Importantly, *Fancd2^–/–^ Lnk^–/–^* mice survived slightly longer than *Fancd2^–/–^* mice, and showed no significant difference in survival compared with WT mice (Figure 2A).

It has been shown that cumulative rounds of replicative stress in WT mice can precipitate an HSC phenotype akin to accelerated aging (33). We directly examined the survival of cohorts of WT, *Fancd2^–/–^*, *Lnk^–/–^*, and *Fancd2^–/–^ Lnk^–/–^* mice during aging. Complete blood counts revealed significantly elevated platelet numbers in aged *Lnk^−/−^* mice, similarly to young mice (9, 10, 14). *Fancd2^−/−^* mice exhibited normal blood counts upon aging, similarly to young mice (14, 34). Notably, aged *Lnk^−/−^* mice showed a significant expansion of white blood cells accompanied by a slight decrease in red blood cell count (Figure 2B), consistent with our previous reports (35). We previously showed that aged *Lnk^−/−^* mice exhibit a myeloproliferative neoplasm–like disease, then progress to and die from monocytic tumors and, to a lesser extent, B cell leukemia (35, 36). In accordance, *Lnk^−/−^* mice died slightly earlier than WT mice (Figure 2C). *Fancd2^−/−^* mice exhibited a similar lifespan to WT mice. Notably, *Fancd2^–/–^ Lnk^–/–^* mice showed reduced myeloid and lymphoid expansion and amelioration of anemia compared with *Lnk^−/−^* mice (Figure 2B), although we were not able to do a comprehensive study of the pathology of the moribund mice. Nonetheless, *Fancd2^−/−^ Lnk*^−/−^ mice showed a similar lifespan to that of *Lnk^−/−^* mice (Figure 2C). Taken together, these data suggest that *Lnk* deficiency moderately promotes survival of *Fancd2^–/–^* mice upon chronic replication stress, and *Lnk* deficiency does not synergize with FA to exacerbate malignancies during aging.

### Lnk deficiency attenuates ATR- but not ATM-mediated DNA damage response upon replication stress.

**Figure 3:**
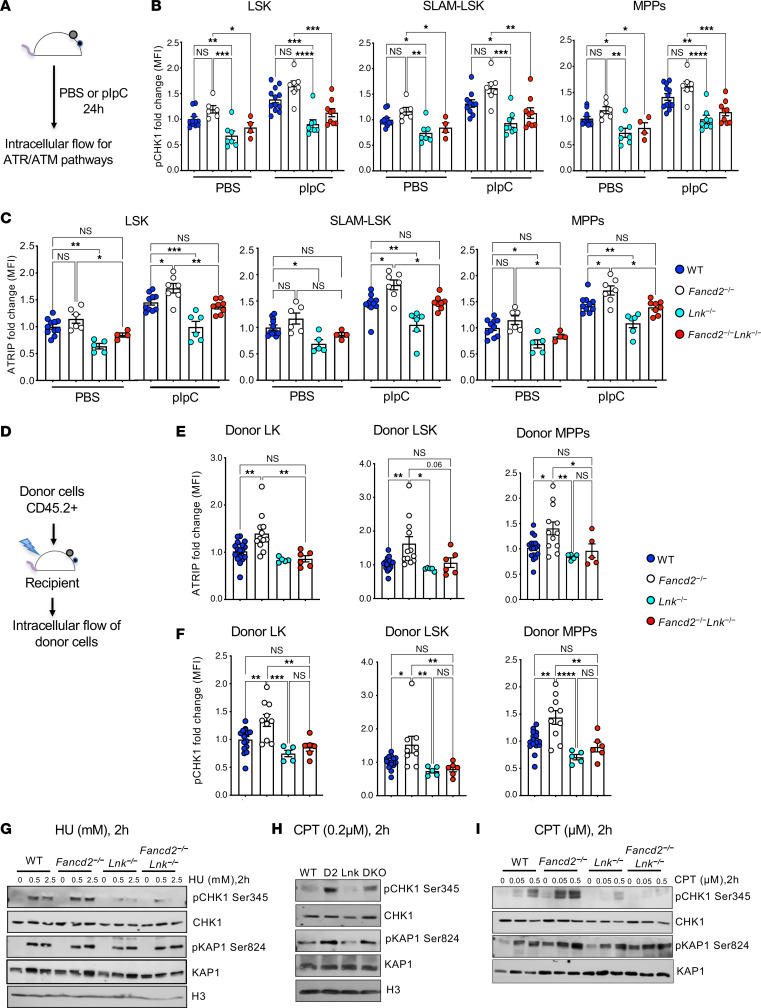
*Lnk* deficiency attenuates the activation of the ATR pathway upon replication stress. (**A**) Experimental design for measuring the activation of the ATR pathway upon pIpC-induced HSPC replication. (**B** and **C**) Quantification of fold change in MFI of pCHK1 (Ser345) (**B**) and ATRIP (**C**) within LSK, SLAM LSK HSC, and MPP populations in mice given PBS or pIpC. (**D**) Experimental design for measuring the activation of the ATR pathway within the donor HSPC population upon transplantation-induced replication. (**E** and **F**) Fold change in MFI of ATRIP (**E**) and pCHK1 (Ser345) (**F**) within donor LK, LSK, and MPP populations after 4 months of BMT. (**G**–**I**) Freshly isolated LK cells from WT, *Fancd2^−/−^* (D2), *Lnk^−/−^* (Lnk), and *Fancd2^−/−^* *Lnk^−/−^* (DKO) mice were treated with the indicated concentrations of HU or CPT for 2 hours in SFEM containing SCF, TPO, IL-3, and IL-6, and the cell lysates were subjected to Western blots for different antibodies. The images in **I** were derived from the same experiment initially shown in Figure 1H (right panel). In all relevant panels, each symbol represents an individual mouse. Bars indicate mean values, and error bars indicate SEM. *P* values were calculated using 1-way ANOVA; *P < 0.05, **P < 0.01, ***P < 0.001, ****P < 0.0001.

DNA damage triggers DNA damage response (DDR), which is coordinated by a network of protein kinase cascades, with ATM and ATR being two major branches. The ATM pathway is primarily activated by double-strand DNA breaks and through checkpoint kinase 2 (Chk2). The ATR pathway is activated by single-strand DNA breaks, and the ATR function is dependent on its binding partner ATRIP, which is recruited to single-stranded DNA upon replication stress and subsequently activates checkpoint kinase 1 (Chk1). Both pathways activate p53 to initiate an array of cellular responses, including cell cycle arrest, apoptosis, checkpoint activation, and DNA repair (37). To examine whether LNK regulates DDR, we evaluated the activation of the ATM and ATR pathways in HSPCs. Since the majority of HSCs are in quiescence or slow in the cell cycle, we administered a single dose of pIpC to mice to induce HSCs into the cell cycle and DNA replication. pIpC increased the percentage of HSCs in the S phase at 24 hours, as previously shown (33) (Supplemental Figure 1; supplemental material available online with this article; https://doi.org/10.1172/JCI191713DS1). It activated both ATM and ATR pathways (Figure 3 and Supplemental Figure 2). *Fancd2^–/–^* HSCs had increased activation of ATR (as indicated by ATRIP and phosphorylated Chk1 [pChk1]) and ATM (as indicated by pATM and pChk2) pathways, compared with WT HSCs. *Lnk* deficiency significantly reduced the activation of the ATR but not the ATM pathway. Importantly, loss of *Lnk* significantly reduced the activation of ATRIP and pChk1 (Figure 3, A–C) but not pATM or pChk2 in the *Fancd2*-null background (Supplemental Figure 2, A and B). We next measured DDR upon transplantation-induced proliferative stress. Total BM cells from different genotypes were transplanted into lethally irradiated mice, and 4 months after BMT, activation of ATRIP, pChk1, pATM, and pChk2 was measured within the donor HSPC population. *Fancd2^–/–^* HSPCs had increased ATRIP/pChk1 activation upon transplantation-induced stress. *Lnk* deficiency significantly decreased ATR activation in *Fancd2^–/–^* HSPCs (Figure 3, D–F). To complement our flow cytometry results by biochemical method to confirm the effect of LNK in regulating the DDR pathways, we treated freshly isolated LK cells with HU or CPT, then subjected these cells to WB analysis with various phospho-specific antibodies against the ATM or ATR pathway effectors. *Fancd2^–/–^* progenitors had increased activation of both ATR (as indicated by pChk1) and ATM (as indicated by p-KAP1) pathways, compared with WT cells. *Lnk* deficiency reduced γH2AX and pChk1 but not p-KAP1 in *Fancd2^–/–^* cells (Figure 3, G–I). Taken together, these data indicate that *Lnk* deficiency attenuates the ATR pathway activation upon replication stress induced by endogenous stressors (pIpC and BMT) as well as exogenous stressors (HU and CPT).

### Lnk deficiency attenuates ATR/p53 checkpoint activation upon replication stress.

**Figure 4:**
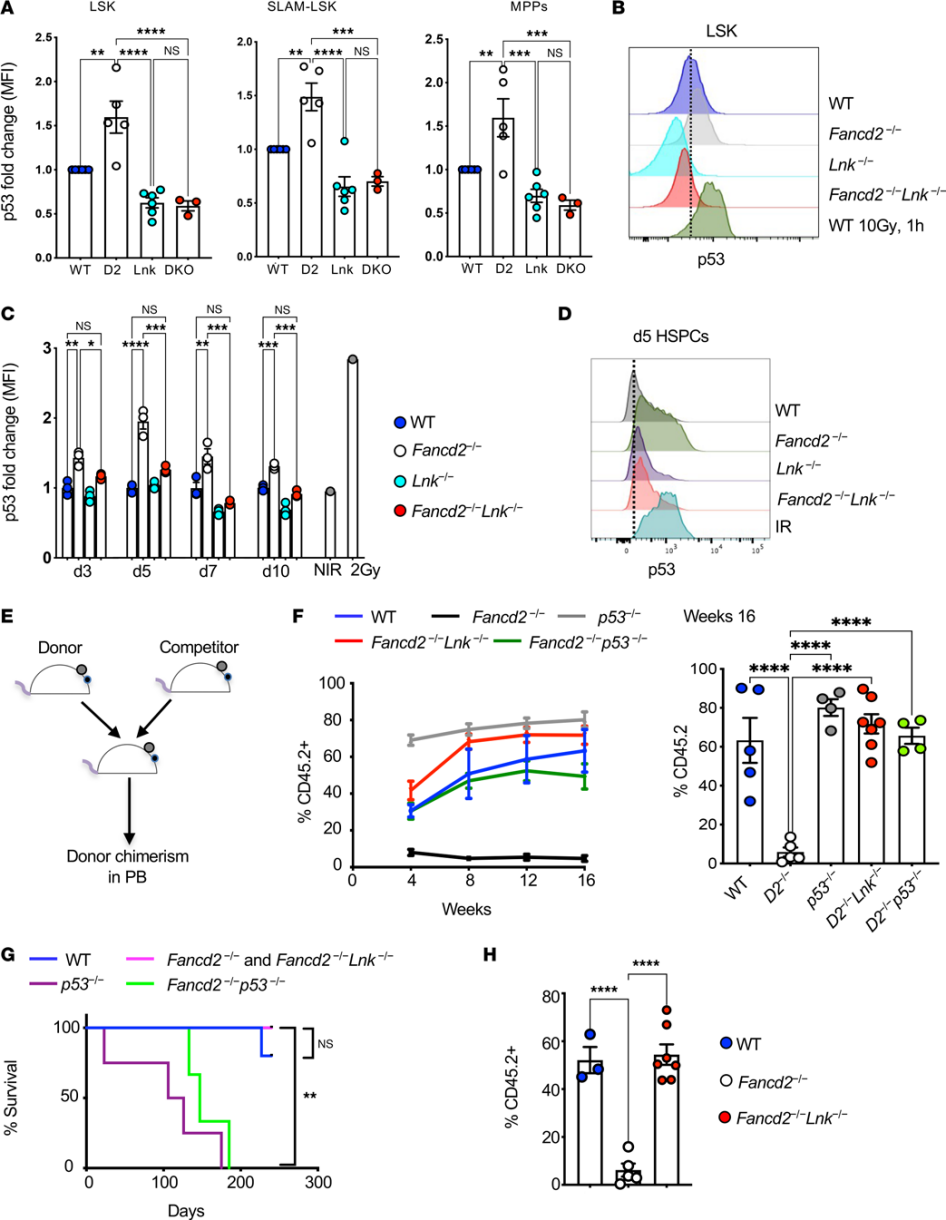
*Lnk* deficiency reduces p53 activation in *Fancd2^–/–^* HSPCs. (**A**) Quantification of p53 levels (fold change in MFI) within LSK cells, SLAM LSK HSCs, and MPPs analyzed by flow cytometry. WT, *Fancd2^−/−^* (D2), *Lnk^−/−^* (Lnk), and *Fancd2^−/−^* *Lnk^−/−^* (DKO) mice are shown. (**B**) Representative histogram plots for p53 in the LSK population. WT mice 1 hour after 10 Gy total-body x-ray irradiation were used as a positive control. (**C**) Quantification of p53 (fold change in MFI) in ex vivo–cultured LSK cells of different genotypes at the indicated days. NIR, non-irradiated. (**D**) Representative histogram plots for p53 in LSK cells. WT cells 2 hours after 2 Gy x-ray irradiation were used as a positive control. IR, irradiated. (**E**) Experimental design for competitive BMT. (**F**) The left panel shows the donor chimerism in the peripheral blood over time, and the right panel shows the percentage of donor reconstitution 16 weeks after transplantation. (**G**) Survival curves of the transplanted mice as in **F**. (**H**) Donor chimerism in the peripheral blood of the recipient mice after 32 weeks. In all relevant panels, each symbol represents an individual mouse. Bars indicate mean values, and error bars indicate SEM. *P* values were calculated using 1-way ANOVA; *P < 0.05, **P < 0.01, ***P < 0.001, ****P < 0.0001.

Upon DNA damage, DDR pathways activate p53, which in turn triggers multiple downstream effects, including cell cycle arrest and apoptosis. Because *Lnk* deficiency reduces the replication stress–induced DNA damage, we sought to investigate the effect of *Lnk* deficiency on p53 activation. p53 was first measured in HSCs at the steady state using flow cytometry. *Fancd2^–/–^* HSCs had significantly increased p53 levels, indicating endogenous stress and elevated DNA damage in unperturbed hematopoiesis. Notably, loss of *Lnk* decreased p53 level in *Fancd2^–/–^* HSCs (Figure 4, A and B). We further analyzed the impact of *Lnk* deficiency in regulating the p53 activation during cellular stress. HSPCs were cultured ex vivo in cytokine-containing medium, and p53 levels were measured at different time points. *Fancd2^–/–^* HSPCs showed significantly higher p53 levels at all the time points, peaking at day 5. Notably, *Lnk* deficiency significantly reduced the p53 levels (Figure 4, C and D). To further examine the effect of *Lnk* in p53 activation during replication stress, fresh LK cells were treated with HU and CPT and then subjected to WB for p53. *Fancd2^–/–^* cells showed increased p53 induction in a dose-dependent manner, while *Lnk* deficiency reduced it (Supplemental Figure 3). These data correlate with the γH2AX levels, suggesting that *Lnk* deficiency reduces the spontaneous DNA damage, thus reducing DDR and p53 activation in FA.

p53 plays a significant role in restricting HSC expansion upon injury (38), and DNA damage–induced p53 is a major reason for HSPC decline in FA (39). The p53 response is activated in *Aldh2^–/–^ Fancd2^–/–^* and *Adh5^–/–^ Fancd2^–/–^* HSCs that have elevated endogenous DNA damage, while p53 deletion rescued this accelerated aging HSC phenotype (40–42). Knockout of p53 also rescued the HSPC defects in *Fanca^–/–^* mice (43). Moreover, p53 silencing improves the HSCs’ function in human FA cells (39). Our previous studies showed that *Lnk* deficiency restores the phenotypic and functional HSCs in *Fand2^–/–^* mice (14). We thus compared the HSC transplant capacity between *Lnk* and p53 deficiency in FA using competitive BMT assays (Figure 4E). Both *Lnk* loss and *p53* loss rescued the BM reconstitution defects of *Fancd2^–/–^* cells, as we did not find a significant difference in BM reconstitution ability between *Fancd2^–/–^ Lnk^–/–^* and *Fancd2^–/–^ p53^–/–^* BM cells (Figure 4, E and F). However, all *p53^–/–^* and *p53^–/–^ Fancd2^–/–^* mice died within 6 months due to T lymphoma (data not shown). In contrast, loss of *Lnk* did not affect the survival of *Fancd2^–/–^* BM-transplanted mice, and the *Fancd2^–/–^ Lnk^–/–^* BM-transplanted mice showed a significantly increased survival rate compared with *p53^–/–^ Fancd2^–/–^* mice (Figure 4G). Notably, loss of *Lnk* in *Fancd2^–/–^* background maintained the BM reconstitution ability for about 8 months (Figure 4H). Taken together, these data indicate that loss of *Lnk* alleviates replication stress–induced DNA damage and ATR/p53 activation, thus improving HSC activity.

### Lnk deficiency reduces chromatin-bound RPA and single-stranded DNA in Fancd2^–/–^ HSPCs.

**Figure 5:**
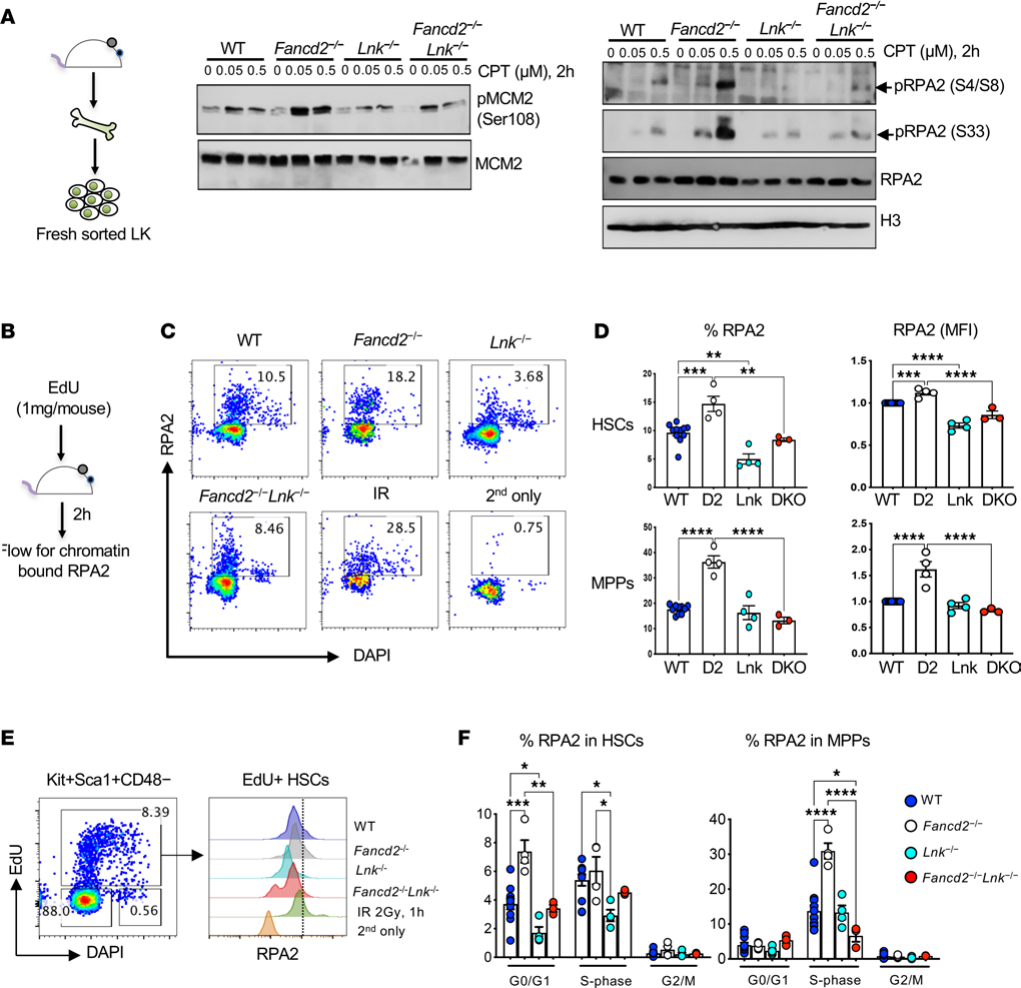
*Lnk* deficiency reduces chromatin-bound RPA in *Fancd2^–/–^* HSPCs. (**A**) Experimental scheme and immunoblots showing the levels of various DDR proteins in freshly sorted LK cells treated with CPT in the presence of cytokines (SCF, TPO, IL-3, IL-6). The images in the middle panel were derived from the same experiment initially shown in Figure 1H (right panel). (**B**–**D**) Comparison of chromatin-bound RPA2 levels in HSPCs upon pIpC-induced replication stress. (**B**) Experimental design. (**C**) Representative flow cytometry plots. IR-induced RPA2 in WT mice was used as a positive control. (**D**) Quantification of the percentage (left panels) and MFI (right panels) of chromatin-bound RPA2 levels in HSC and MPP populations. WT, *Fancd2^−/−^* (D2), *Lnk^−/−^* (Lnk), and *Fancd2^−/−^* *Lnk^−/−^* (DKO) mice are shown. (**E**) Representative flow cytometry plot showing different cell cycle stages and histogram plot for chromatin-bound RPA2 within EdU^+^ HSCs (S phase) from different mouse groups. (**F**) Percentages of chromatin-bound RPA2^+^ cells in different cell cycle stages of HSCs and MPPs. In all relevant panels, each symbol represents an individual mouse. Bars indicate mean values, and error bars indicate SEM. *P* values were calculated using 1-way ANOVA; *P < 0.05, **P < 0.01, ***P < 0.001, ****P < 0.0001.

ATR signaling is activated by the recruitment of replication protein A (RPA) to single-stranded DNA (ssDNA) generated through the uncoupling of MCM helicases that continue to uncoil the DNA strand ahead of the slowed/stalled DNA polymerases upon replication stress (27). Because loss of *Lnk* attenuates the ATR pathway activation upon replication stress, we hypothesize that *Lnk* deficiency reduces ssDNA breaks that trigger the phosphorylation of ATR and its substrates. Freshly isolated LK cells were treated with increasing concentrations of CPT or HU and then subjected to WB (Figure 5A). As previously reported in cell lines (44), loss of *Fancd2* induced the phosphorylation of pSer33-RPA2 and pSer108-MCM2, markers of replication stress known to be phosphorylated by ATR, in primary HSPCs. Notably, loss of *Lnk* reduced their phosphorylation in *Fancd2^–/–^* cells. Persistent replication stress leads to severe DNA damage and ATM activation. Indeed, *Lnk* deficiency also attenuated pSer4/8-RPA2, which is a known ATM substrate (Figure 5A and Supplemental Figure 4A).

We next measured the accumulation of RPA2 on the chromatin of the nascent DNA strand from endogenous stress in vivo. We injected EdU into mice 2 hours before BM harvesting, and the chromatin-bound RPA2 levels were measured in the HSC and multipotential progenitor (MPP) populations by flow cytometry (Figure 5B and Supplemental Figure 4B). As expected, MPPs exhibited a higher percentage of RPA2^+^ cells than HSCs, since MPPs are more proliferative than HSCs. A significantly increased RPA2 level was observed in both HSCs and MPPs of *Fancd2^–/–^* mice. Notably, loss of *Lnk* significantly reduced the RPA2 accumulation in both HSCs and MPPs (Figure 5, C and D). During replication stress, RPA2 is recruited to and binds ssDNA generated by stressed or stalled replication forks; thus it peaks at the S phase. To further dissect how the loss of *Lnk* regulates the RPA2 recruitment at the replication fork, we quantified RPA2 levels in different phases of the cell cycle (Figure 5E). In MPPs, chromatin-bound RPA2 reached the highest level in the S phase, with *Fancd2^–/–^* mice showing a significant increase in comparison with WT. Interestingly, in HSCs, *Fancd2^–/–^* cells had elevated RPA2 levels in the G_0_/G_1_ phase. Notably, *Lnk* deficiency reduced the RPA2 accumulation in both HSCs and MPPs in different cell cycle stages (Figure 5F).

**Figure 6:**
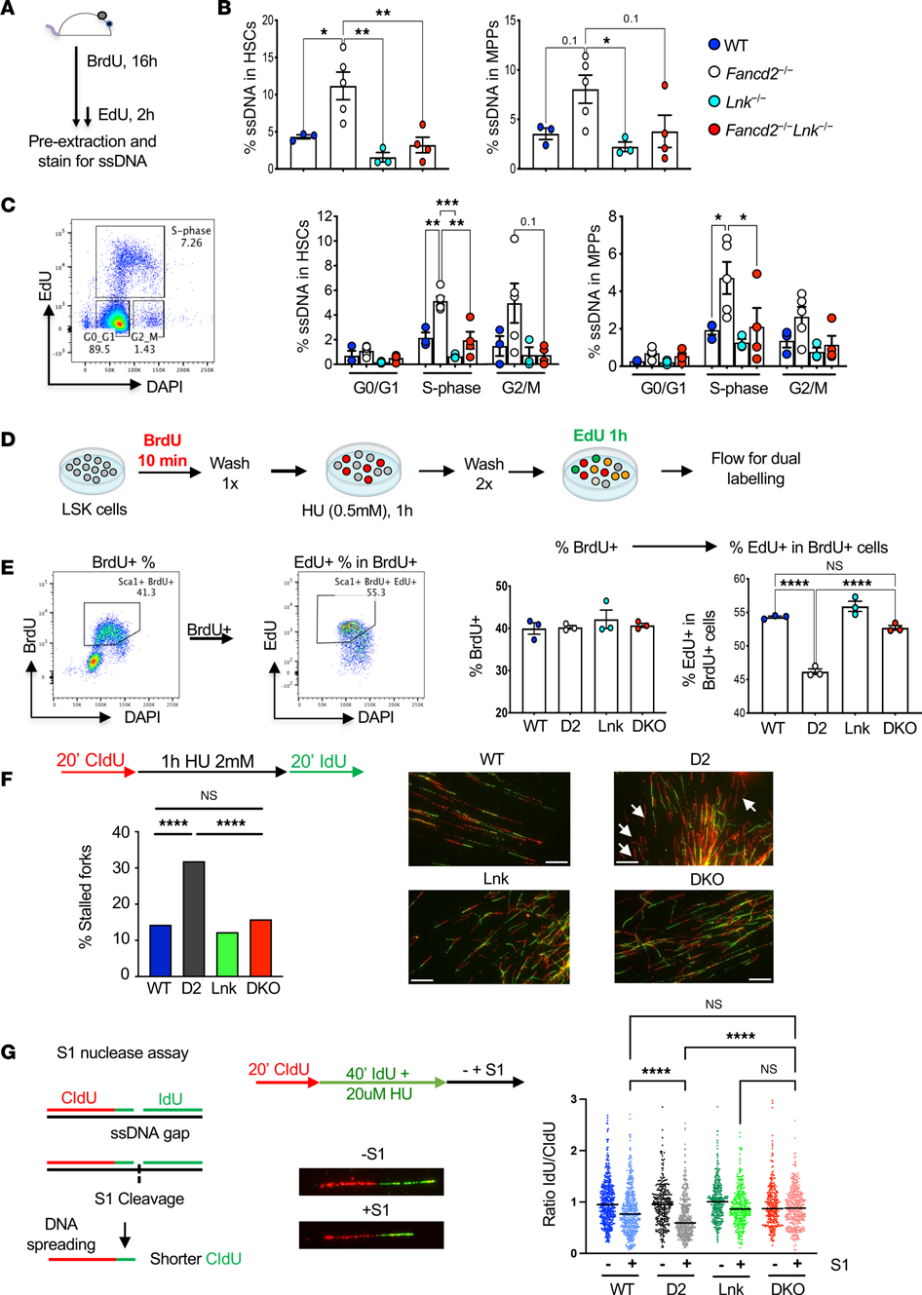
*Lnk* deficiency reduces ssDNA breaks and promotes replication fork recovery at stalled replication forks in *Fancd2^–/–^* HSPCs. (**A**) Experimental design of the procedure to measure ssDNA in vivo. (**B**) Quantification of total ssDNA within HSCs and MPPs. (**C**) Representative flow plot showing cell cycle stages and percentage of ssDNA in different cell cycle phases of HSCs and MPPs. (**D**) Schematic outline of the experimental procedure for fork restart assay by flow cytometry. (**E**) Cultured LSK HSPCs from WT, *Fancd2*^−/−^ (D2), *Lnk*^−/−^ (Lnk), and *Fancd2*^−/−^ *Lnk*^−/−^ (DKO) mice were subjected to the fork restart assay. Left: Representative flow plots of the restarted replication upon HU-induced fork stalling in cultured LSK HSPCs. Right: Percentages of EdU^+^ cells from BrdU^+^ cells of cultured HSPCs. (**A**–**E**) In all relevant panels, each symbol represents an individual mouse. Bars indicate mean values, and error bars indicate SEM. *P* values were calculated using 1-way ANOVA; *P < 0.05, **P < 0.01, ***P < 0.001, ****P < 0.0001. (**F**) Freshly isolated primary LSK cells were subjected to fork recovery assay upon HU-mediated replication stalling using single-molecule DNA fibers. Top left: Experimental overview of the assay. Right: Representative images of DNA fibers. White arrows indicate stalled replication forks (red only); recovered forks show red-green tracks. The frequencies of the stalled replication forks upon high-dose HU are plotted. One hundred seventy-five to 300 fibers were analyzed per group. Similar results were obtained from 2 biological repeats. Statistical significance by Fisher’s exact test is shown. Scale bar: 20 μm. (**G**) Experimental scheme and representative DNA fibers from the D2 group are shown for the S1 nuclease assay to examine ssDNA fibers. Fresh LSK cells from WT, *Fancd2^−/−^* (D2), *Lnk^−/−^* (Lnk), and *Fancd2^−/−^* *Lnk^−/−^* (DKO) mice were pulsed with CldU for 20 minutes followed by IdU in the presence of low-dose HU for 40 minutes. Right: Ratios of IdU to CldU DNA fibers; horizontal lines indicate geometric mean. Two hundred to 500 fibers were analyzed per group. *P* values were calculated using Kruskal-Wallis test; ****P < 0.0001. Similar results were obtained from 2 biological repeats.

The elevated RPA2 level in the G_0_/G_1_ phase of *Fancd2^–/–^* HSCs could be due to unrepaired DNA damage in the G_2_/M phase of the previous cell cycle. We thus directly assessed the proportion of cells with ssDNA generated from endogenous replication stress in vivo. Sixteen-hour BrdU labeling coupled with antibody staining under non-denaturing conditions was used to measure the ssDNA on the nascent DNA strands. Two hours of EdU labeling at the end of BrdU labeling allowed us to evaluate the ssDNA generated in different phases of the cell cycle (Figure 6A and Supplemental Figure 5A). Replication stress induced by pIpC or IR increased the proportion of ssDNA, as expected (Supplemental Figure 6A). *Fancd2^–/–^* HSCs had a higher proportion of cells with ssDNA compared with WT, and this difference was the most pronounced in the S phase (Figure 6, B and C). Importantly, loss of *Lnk* significantly reduced ssDNA percentage in *Fancd2^–/–^* HSCs (Figure 6, A–C). Together, these data suggest that *Lnk* deficiency reduces chromatin-bound RPA and ssDNA breaks at the replication forks, leading to reduced ATR activation during endogenous stress.

### Lnk deficiency suppresses ssDNA gaps and promotes replication fork recovery at the stalled forks in Fancd2^–/–^ HSPCs.

Unrepaired DNA breaks delay or prevent replication restart once DNA damage reagents are removed; thus, we examined replication restart upon stress. Cultured HSPCs were pulsed with BrdU to label nascent DNA at the replication fork and then subjected to HU for 1 hour to induce replication fork stalling and halt S-phase progression (Supplemental Figure 5B). We then washed off HU and labeled cells that were re-entering the S phase with EdU (Figure 6D). BrdU^+^ cells are measured as cells present in the first cell cycle, while BrdU^+^EdU^+^ cells are measured for the efficiency of replication fork restart (Figure 6E). There was no significant difference in the BrdU incorporation in different groups of HSPCs, suggesting a comparable proliferation rate among the groups in the short-term culture. However, *Fancd2^–/–^* HSPCs had reduced percentage EdU^+^ within the BrdU^+^ population, indicating a significant defect in replication restart upon HU treatment. Importantly, *Lnk* loss significantly overcame this defect and restored replication restart in *Fancd2^–/–^* HSPCs to WT levels (Figure 6E).

To directly examine fork stalling versus fork recovery upon replication stress, we subjected freshly isolated LSK cells to a fork recovery assay in single-molecule DNA fiber analysis (45, 46). LSK cells were pulse-labeled with IdU (5-Iodo-2′-deoxyuridine) and then subjected to high-dose HU-mediated replication fork stalling. We then washed off HU and pulsed the cells with CldU (5-Chloro-2′-deoxyuridine). Single DNA fibers were spread onto microscope slides before immunofluorescence staining with antibodies against CldU and IdU to quantify stalled (IdU or red only) or recovered (IdU-CldU or red-green) forks. *Fancd2^–/–^* HSPCs had significantly increased stalled forks (Figure 6F). *Lnk* loss significantly overcame this defect and restored replication restart in *Fancd2^–/–^* HSPCs to WT levels (Figure 6F).

To directly measure ssDNA gaps in freshly isolated LSK cells, we performed the S1 nuclease assay using single-molecule DNA fibers (Figure 6G). We examined HSPC replication under mild replication, i.e., low-dose HU that slows but does not halt replication. Replication tracts of LSK cells were sequentially pulse-labeled with IdU followed by CldU in the presence of low-dose HU. Nuclei were then isolated and split into no-S1 and S1-treated groups. Single DNA fibers were spread onto microscope slides before immunofluorescence staining with antibodies against IdU and CldU. Since S1 nuclease only cleaves ssDNA, the CldU length is used to quantify intact or cleaved DNA fibers, serving as a measure of ssDNA gaps (46). All 4 groups of HSPCs show similar fiber lengths without S1 treatment, indicating similar fork speeds. Upon S1 treatment, *Fancd2^–/–^* HSPCs exhibited a significant reduction in replication fork length, indicating elevated ssDNA gaps in mild replication stress. Importantly, *Lnk* deficiency significantly suppressed ssDNA gaps in *Fancd2^–/–^* HSPCs (Figure 6G). Thus, our data suggest that *Lnk* deficiency promotes replication fork recovery at the stalled replication forks in high stress and reduces ssDNA gaps and facilitates replication in *Fancd2^–/–^* HSPCs under endogenous or mild stress. Taken together, our data suggest that *Lnk* deficiency reduces ssDNA breaks and promotes replication fork recovery at the stalled replication forks in *Fancd2^–/–^* HSPCs.

### The superior reconstituting activity of Lnk^–/–^ HSCs depends on REV1-mediated TLS.

**Figure 7:**
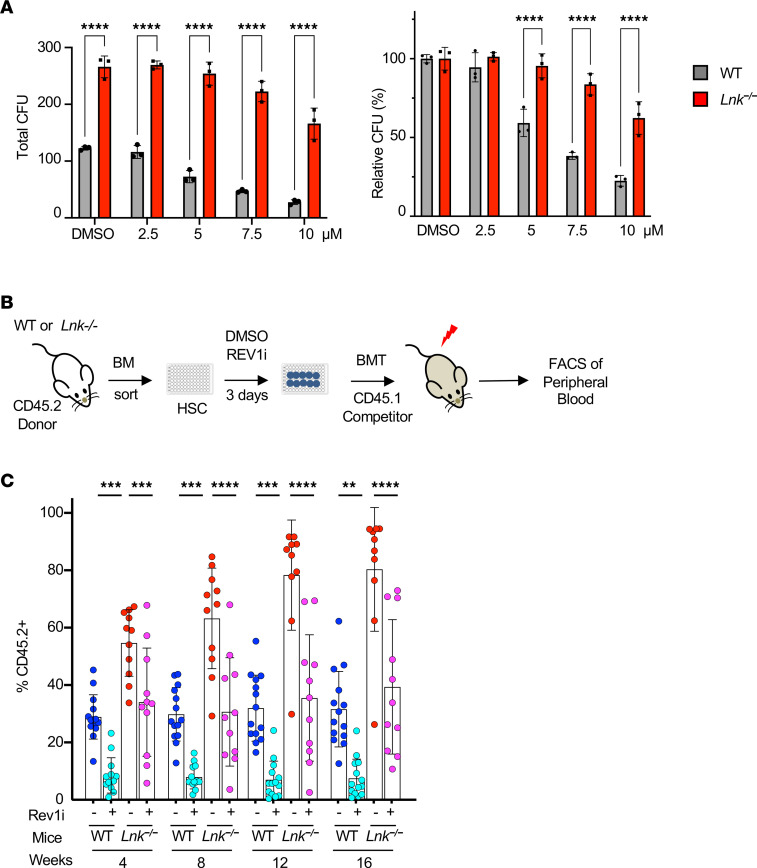
*Lnk*-deficient HSCs are sensitive to REV1 inhibition, and their superior reconstituting activity depends on REV1-mediated TLS. (**A**) BM cells from WT and *Lnk^–/–^* mice were plated in semi-solid methylcellulose culture containing a graded dose of REV1i in triplicate, at 30,000 and 15,000 cells per plate, respectively. Total (left) and relative (right) colony-forming capacities are shown. Mean values and SD are shown. *P* values were calculated using 2-way ANOVA; ****P < 0.0001. (**B**) Schematic illustration of the lentiviral transduction/BMT experimental scheme. (**C**) SLAM LSK HSCs from WT and *Lnk^–/–^* mice were sorted into 96-well plates. Cells were cultured in DMSO or 5 μM REV1i for 3 days; then cells from each well were transplanted into each lethally irradiated recipient mouse. Donor percentages from each group in the peripheral blood are shown. Each symbol represents an individual mouse. Horizontal lines indicate mean frequencies, and error bars indicate SD. *P* values were calculated using 1-way ANOVA; **P < 0.01, ***P < 0.001, ****P < 0.0001.

To examine the mechanisms by which *Lnk* loss ameliorates replication-associated DNA damage, we turned to TLS, one of the DNA damage tolerance (DDT) mechanisms that utilize specialized DNA polymerases to bypass DNA lesions or facilitate replication through replication fork impediments, thus preventing the stalling of DNA replication and the exacerbation of DNA damage (20). TLS is mediated by PCNA monoubiquitination or REV1-dependent pathways (20). While REV1 has a catalytic role in TLS, its major role is to act as a chaperone to recruit other TLS polymerases, such as Polη, Polι, and Polκ, to the sites of DNA stress and to facilitate switching between the inserter DNA polymerase and the extender DNA polymerase Polζ. The REV1-dependent recruitment of Polη to stalled forks is particularly important for “on-the-fly” TLS upon replication barriers (32, 47). Therefore, we first examined the sensitivity of *Lnk*-deficient HSPCs to REV1 inhibition using a small-molecule inhibitor, REV1i (JH-RE-06), that disrupts its ability to recruit Polζ (48). We plated WT and *Lnk^–/–^* BM cells in semi-solid methylcellulose cultures containing a graded dose of REV1i. While REV1 inhibition reduced the clonogenic ability of both WT and *Lnk^–/–^* BM cells, *Lnk* deficiency conferred reduced sensitivity to REV1i (Figure 7A). We next examined HSC repopulation ability upon REV1 inhibition. HSCs (CD150^+^CD48^–^LSK or SLAM LSK cells) from WT and *Lnk^–/–^* mice were sorted into 96-well plates and treated with 5 μM REV1i or DMSO vehicle control for 3 days. We then transplanted each well into each lethally irradiated recipient mouse (Figure 7B). HSCs appear to be more sensitive to REV1i than progenitors. REV1i significantly reduced the expansion and repopulation ability of both WT and *Lnk^–/–^* HSCs (Figure 7C). Notably, REV1i reduced *Lnk^–/–^* HSCs to a level similar to that of WT HSCs (Figure 7C). This suggests that the superior reconstituting activity of *Lnk^–/–^* HSCs depends on REV1-mediated TLS.

### Lnk-deficient HSCs have increased chromatin-bound Polƞ, but their superior reconstituting activity largely does not depend on Polƞ alone.

**Figure 8:**
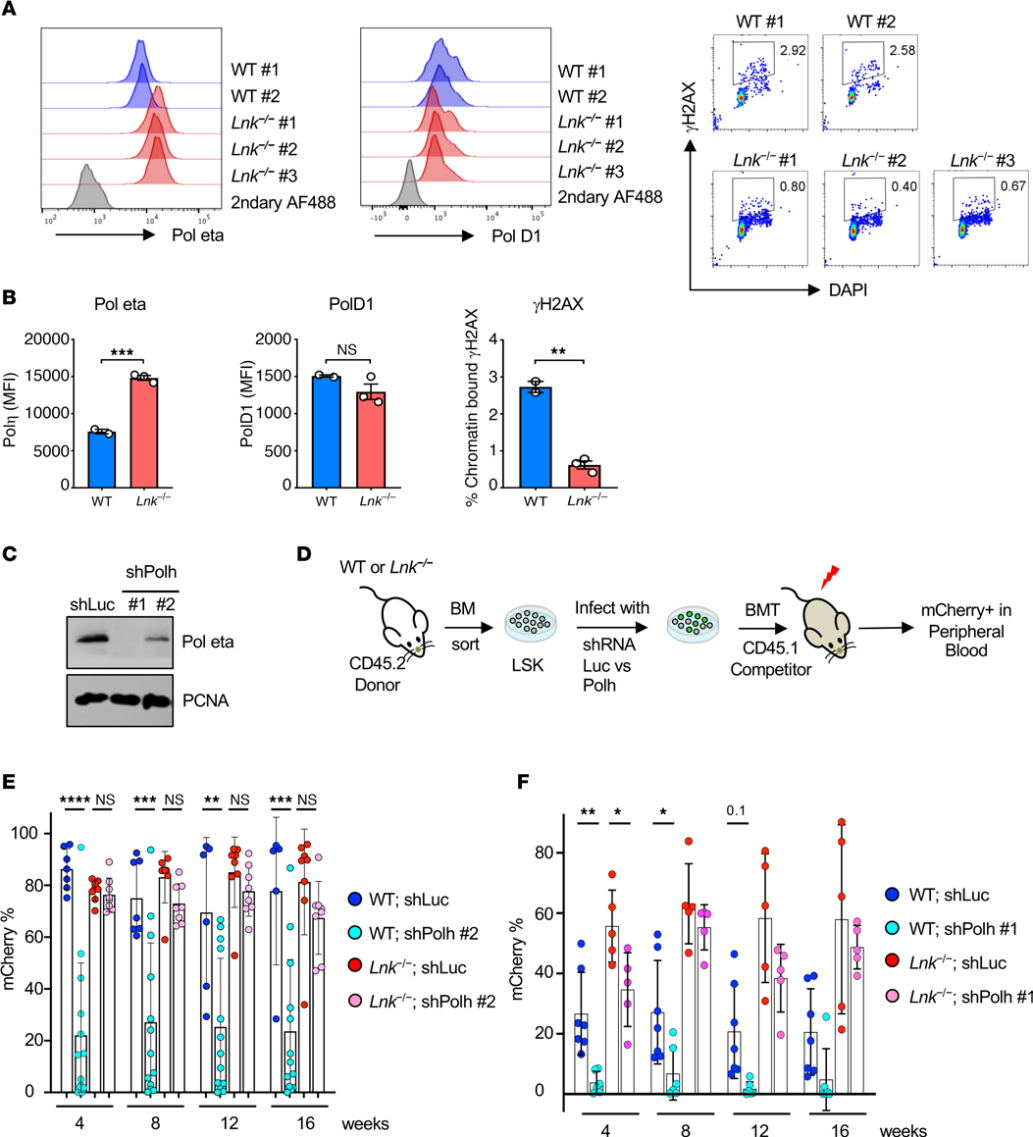
*Lnk-*deficient HSCs have increased chromatin-bound Polƞ, and their superior reconstituting activity in part depends on Polƞ. (**A**) Representative flow cytometric histogram plots for chromatin-bound Polη (left) and Polδ1 (D1, middle) in the HSCs of WT and *Lnk*^−/−^ mice. γH2AX/DAPI flow plots are shown (right). (**B**) Quantification of MFIs of Polƞ, Polδ1, and γH2AX in HSCs as examined in **A**. (**C**) Examination of the efficiency of Polƞ depletion using 2 different shRNAs by WB. (**D**–**F**) LSK cells from WT and *Lnk^−/−^* mice were infected with lentiviruses expressing shRNA-mediated knockdown of *PolH* or luciferase (Luc) as a control and subsequently transplanted into lethally irradiated recipient mice. (**D**) Schematic illustration of the lentiviral transduction/BMT experimental scheme. (**E** and **F**) Quantification of mCherry^+^ percentages within CD45.2^+^ donors in the peripheral blood from each group after transplantation using shRNA-PolH#2 (**E**) or shRNA-PolH#1 (**F**). Each symbol represents an individual mouse. Bars indicate mean frequencies, and error bars indicate SD. *P* values were calculated using 1-way ANOVA; *P < 0.05, **P < 0.01, ***P < 0.001.

REV1 recruits multiple specialized TLS DNA polymerases, such as Polη, that play pivotal roles in mitigating a wide range of DNA replication impediments during DNA replication. Examination of our prior genome-wide gene expression data (13) revealed that the mRNA expression level of various DNA polymerases is unchanged in *Lnk^–/–^* HSCs compared with WT HSCs. We thus examined the protein level of TLS polymerases in *Lnk^–/–^* HSCs. Because of the antibodies’ availability for intracellular flow cytometry, we examined Polη and Polδ1. We found that *Lnk^–/–^* HSCs had increased chromatin-bound Polη but not Polδ1 (a replicative DNA polymerase), implicating their enhanced TLS activity through Polη (Figure 8, A and B). To test that, we assessed whether HSCs are sensitive to *PolH* depletion in vivo using the viral infection/BMT model using 2 different shRNAs to *PolH* (Figure 8, C–F). Sorted LSK cells from WT and *Lnk^–/–^* mice were infected with lentiviruses expressing microRNA-30–based shRNA against *PolH* or luciferase (Luc) with mCherry as a marker, followed by transplantation into lethally irradiated recipient mice (Figure 8D). The 4 experimental groups had similar infection rates at the time of transplantation, as shown by percentage mCherry^+^ cells (Supplemental Figure 6). We quantified percentage mCherry^+^ cells in the donor population of the peripheral blood of recipient mice to examine HSC activity in vivo. *PolH* depletion in WT HSCs significantly reduced stem cell repopulating ability; in contrast, *Lnk^–/–^* HSCs were more resistant to *PolH* depletion by both shRNAs, exhibiting reconstitution ability superior to that of WT HSCs (Figure 8, E and F). Notably, the stronger *PolH* depletion via sh*PolH*#1 moderately reduced *Lnk^–/–^* HSCs, but only in the short-term transplants (Figure 8F). REV1 recruits other TLS polymerases, such as Polι and Polκ, in addition to Polη, implicating redundant roles of different TLS polymerases in mediating the superior ability of *Lnk^–/–^* HSCs to mitigate replication stress during regeneration.

## Discussion

The adaptor protein LNK is a critical negative regulator of HSC expansion (7, 10–12, 49). We previously showed that loss of *Lnk* restores mouse and human FA HSPCs by alleviating replication stress (14, 15). Understanding LNK regulatory functions in promoting HSC fitness will likely reveal fundamental aspects of stem cell biology. This study uncovers a role for LNK in regulating TLS polymerases to control HSC expansion. *Lnk* deficiency mitigates replication-associated DNA damage. This phenotype is more pronounced in the FA background, even in the absence of exogenous DNA damage insults. We show that *Lnk* deficiency suppresses ssDNA breaks and improves replication fork restart, leading to reduced ATR/p53 checkpoint activation that is linked to HSC attrition. Mechanistically, enhanced HSC fitness due to *Lnk* deficiency is associated with increased TLS activity via REV1 (Supplemental Figure 7). The mechanisms by which *Lnk* inhibition alleviates endogenous replication stress and promotes HSC fitness have important ramifications for other types of BM failure syndromes as well as stem cell therapy in general.

This work uncovers that HSPCs utilize TLS DNA polymerases to help alleviate replication stress and promote stem cell fitness. Our data suggest that REV1 and, to a lesser extent, Polη activities contribute to the enhanced HSC fitness observed in *Lnk^–/–^* HSCs. There are multiple overlapping but distinct TLS pathways and multiple TLS polymerases; thus, inhibiting one arm of the TLS pathways does not completely block TLS activity. We hypothesize that *Lnk* deficiency has enhanced overall TLS activity, which makes *Lnk*-deficient HSPCs less sensitive to partial TLS inhibition, i.e., Polη knockdown. Moreover, our data do not exclude other TLS-independent mechanisms of mitigation of replication stress by *Lnk* deficiency. *Polh^−/−^* mice display no overt defects, except alterations in antibody hypermutation in B cells (50). XP-V patients with *POLH* mutations are not known to develop BM failure or leukemia (51, 52). HSPC phenotypes in *Polh^−/−^* mice have not been reported. *Rev1^–/–^* mice are infertile but grossly normal. *Rev1^–/–^* mice showed compromised BMT ability, although it was examined on a mixed background (23). These germline knockout mice may experience compensation during hematopoietic development; thus, HSPC phenotypes would only be manifested upon stress. Our data using shRNA depletion or acute pharmacological inhibitor in adult BM HSCs, subjecting HSCs to culture and transplantation-associated stress, may account for a more pronounced phenotype. REV1 and Polη function in overlapping but distinct TLS pathways, and REV1 recruits other TLS polymerases such as Polι and Polκ, in addition to Polη; thus, reducing TLS through one of them may not completely negate the HSC fitness associated with *Lnk* deficiency. Therefore, it will be important to formally interrogate the potential redundant roles of REV1, Polη, and other TLS polymerases in regulating HSC regenerative activity and upon stress in mouse models.

TLS is a type of DDT mechanism that enables specialized TLS polymerases to continue replication in the presence of lesions that otherwise block the replicative polymerases Polδ and Polε. Continued replication, although with an increased error rate, is considered to be better than prolonged fork stalling and fork collapse, which are associated with deleterious DNA damage and structural changes (deletion, insertion, rearrangement), thus genome instability. Recent studies reveal the non-canonical role of TLS polymerases, in particular Polη, that play important roles in DNA synthesis at undamaged DNA but difficult-to-replicate regions of the genome such as common fragile sites; the generation of immunoglobulin diversity during somatic hypermutation in memory B cells; processing of R-loops during replication-transcription conflict; and maintenance of telomere length through the alternate lengthening of telomeres (ALT) pathway (32). These canonical and non-canonical functions of TLS are crucial mechanisms to protect the genome.

DDT pathways also include template switching, fork reversal, and repriming, besides TLS. K^164^-monoubiquitination of PCNA upon DNA lesions is the molecular trigger to switch from the replicative polymerase to the specialized TLS polymerases, to allow the replisome to progress while tolerating DNA damage. Disruption of PCNA K^164^-monoubiquitination reduces the recruitment of multiple TLS polymerases. *Pcna^K164R^* mice show a strong HSC transplant defect, reminiscent of premature aging and stressed hematopoiesis (24). Notably, PCNA is involved in multiple DDT mechanisms; K^164^-monoubiquitination is involved in TLS, while K^164^-polyubiquitination is involved in template switching (20), both of which contribute to K^164^-PCNA’s role in HSPCs. Studies of mice deficient in PrimPol, a specialized DNA polymerase for repriming, revealed the requirement of PrimPol for efficient HSC amplification and BM reconstitution (53). DDT warrants continuation of replication through bypassing the impediment to replicate without gaps or repriming downstream of the impediment, then filling ssDNA gaps after replication (20). TLS polymerases are increasingly being recognized as key players in all DDT mechanisms to mitigate replication stress, critically contributing to genome stability and cellular fitness (21). Overall, the role of the complex DDT pathways in HSCs emphasizes a need for future investigations.

We previously showed that LNK directly interacts with phosphorylated JAK2 and dampens TPO/MPL/JAK2 signaling in controlling HSC self-renewal (12). Our data in this report suggest that LNK directly or indirectly impacts TLS. However, *Lnk* deficiency may also influence alternative DNA repair pathways. Indeed, TPO was shown to increase DNA repair efficiency in HSCs through an enhanced non-homologous end joining (NHEJ) mechanism upon IR (54, 55). Thus, increased NHEJ and/or homologous recombination in the absence of *Lnk* may help FA or TLS*-*deficient HSC survival. It remains to be formally tested whether LNK plays a role in other types of DNA repair in physiologically relevant cells. LNK is a cytoplasmic adaptor protein that is not known to associate with chromatin (12, 36, 56, 57). However, we cannot exclude the possibility that a small fraction of LNK proteins exert functions in the nucleus. The elucidation of how LNK impacts TLS and the recruitment of TLS polymerases to the stressed replication forks is a subject of ongoing investigation.

Our results suggest that enhancing TLS activity could increase HSC functions by reducing replication-associated DNA damage. One concern regarding enhancement of TLS activity is its error-prone nature in replication through non-damaged DNA templates. Whole-genome sequencing of cells lacking components of TLS, such as REV1 or PCNA-Ub, or PrimPol, suggests that TLS and DDT in general protect the genome from deletions and large rearrangements at the expense of spontaneous base substitutions (58). TLS contributes to genetic variance in the human genome. Future studies should conduct in-depth investigations of potential genomic alterations/mutations affected by enhancement of TLS activity. Nonetheless, our work sheds light on amelioration of replication stress to promote HSC fitness in healthy and disease settings. It also serves as the basis for the future development of small molecules to modulate TLS activities for stem cell therapy. It is important to note that *Lnk* deficiency reduces DNA damage–mediated ATR/p53 checkpoint activation, preventing HSPC attrition in FA mice upon acute and chronic replication stress. This remarkable ability contrasts with the loss of *p53*, which was also shown to improve HSC activity in FA. Loss of the tumor suppressor *p53* is associated with carcinogenesis and lymphoma in mice. Notably, we show that the combined loss of *Lnk* and *Fancd2* does not reduce survival or increase hematologic malignancies of mice with single *Lnk* knockout during aging. Understanding mechanisms by which *Lnk* deficiency mitigates replication stress and suppresses DNA damage–induced HSC attrition will facilitate the development of strategies that have the dual benefit of enhancing HSC fitness and simultaneously reducing genome instability.

## Methods

### Sex as a biological variable.

Our study examined male and female animals, and similar findings are reported for both sexes.

### Mice.

*Fancd2*^−/−^ mice were generously provided by Alan D’Andrea (Dana-Farber Cancer Institute, Boston, Massachusetts, USA) (34), and *Lnk*^−/−^ mice were obtained from Tony Pawson (Samuel Lunenfeld Research Institute, Toronto, Ontario, Canada) (10). *Tp53*^−/−^ mice (stock 002101) were purchased from The Jackson Laboratory (59). Both male and female mice (2–6 months old) were used in this study. SJL (CD45.1) mice were purchased from The Jackson Laboratory and bred in our facilities. SJL (CD45.1) mice were crossed with C57BL/6J (CD45.2) mice to generate CD45.1/CD45.2 F_1_ mice. All the animal studies were conducted under a protocol approved by the Institutional Animal Care and Use Committee of the Children’s Hospital of Philadelphia.

### Hematology and flow cytometry of HSPCs and lineage cells.

Peripheral blood was collected from the retro-orbital sinus in heparinized tubes. Complete blood count was measured using a Hemavet 950 (Drew Scientific). For lineage analysis by FACS, blood cells were lysed using RBC lysis buffer (StemCell Technologies Inc.) and stained with different fluorochrome-conjugated anti-CD45.1 (A20), -CD45.2 (104), –Gr-1 (RB6-8C5), -Mac1 (M1/70), -CD19 (eBio1D3), and -CD3 (17A2) antibodies (from Biolegend or eBiosciences). Lineage samples were stained with PI, and acquisition was performed using a FACSCanto II flow cytometer (BD Biosciences). All the data were analyzed using FlowJo.

HSPC staining was conducted as described previously (60). Briefly, cells were quickly lysed with RBC lysis buffer and then stained with biotin-conjugated anti–Gr-1 (RB6-8C5), -Mac1 (M1/70), -B220 (RA3-6B2), -CD19 (eBio1D3), -Ter119 (TER-119), -CD5 (53-7.3), -CD4 (GK1.5), and -CD8 (53-6.7) antibodies, in combination with APC–Cy7–c-Kit (2B8), PerCP-Cy5.5-Sca1 (E13-161.7 or D7), FITC-CD48 (HM48-1), and PE-Cy7-CD150 (TC15-12F12.2), for 30 minutes on ice (all from Biolegend or eBiosciences unless otherwise noted), followed by secondary staining with streptavidin–PE–Texas red (1:50; Invitrogen SA1017). Different HSPC subpopulations were defined as HSCs (Lin^–^Sca1^+^c-Kit^+^Flk2^–^CD150^+^CD48^–^) and MPPs (CD150^–^CD48^+^LSK) (61). Cells were resuspended in DAPI-containing buffer and subjected to flow analysis on a FACS Fortessa (BD Biosciences) or Cytek Aurora flow cytometer.

### Flow cytometry of intracellular antigens.

Briefly, single-cell suspensions of BM cells were obtained from the hind limb together with pelvic bones. Cells were enriched for lineage negative (Lin^–^) using the Lineage Cell Depletion Kit (Miltenyi Biotec). Bead-labeled cells were separated by autoMACS Pro (Miltenyi Biotec) according to the manufacturer’s instructions. Lin^–^ cells were first stained with Live/Dead Fixable Aqua (Invitrogen) followed by surface staining for HSPCs. After surface staining, cells were fixed with Cytofix/Cytoperm buffer (BD Biosciences) for 20 minutes at 4°C, followed by washing with 1× Perm/Wash buffer (BD Biosciences). Cells were further incubated with Permeabilization Buffer Plus (BD Biosciences) for 10 minutes at 4°C. After washing with 1× Perm/Wash buffer, cells were further incubated with intracellular antibodies for 2 hours at room temperature in the dark, with anti-pCHK1 (1:200; Invitrogen MA5-15145), anti-ATRIP (1:200; Invitrogen PA1-519), PE–anti-pATM (Ser1981) (1:100; BioLegend 651204), PE–anti-pCHK2 (Thr68) (1:100; BioLegend 12-9508-42), AF488-γH2AX (1:100; BioLegend 613406), and AF488-p53 (1:50; Cell Signaling Technology 2015S). For the unconjugated antibodies, cells were further incubated with rabbit AF647 secondary antibody (1:250; Invitrogen A-21244) for 30 minutes at room temperature. After washing, cells were resuspended in 1× Perm/Wash buffer and acquired on a BD Fortessa flow cytometer.

For the detection of chromatin-bound proteins, 5-ethynyl-2′-deoxyuridine (EdU; 1 mg) was injected into the mice i.p. for 2 hours. Mice were sacrificed, and c-Kit^+^ cells were isolated using CD117 microbeads (Miltenyi Biotec) and stained with PerCP-Cy5.5-Sca1 (D7) and APC-Cy7-CD48 (HM48-1) cell surface markers for 30 minutes at 4°C. For pre-extraction, cells were washed once with PBS and incubated with ice-cold pre-extraction buffer (PBS containing 0.2% Triton X-100) for 1 minute on ice before fixation. Fixation was performed using Cytofix/Cytoperm buffer (BD Biosciences) for 20 minutes at 4°C. Cells were permeabilized using 1× Perm/Wash buffer (BD Biosciences). Click-iT reaction was performed according to the manufacturer’s protocols using AF647 azide, triethylammonium salt (A10277) to stain EdU. For the intracellular staining, cells were incubated for 2 hours at room temperature with anti-RPA2 (1:100; Abcam ab76420), AF488-γH2AX (1:100; BioLegend 613406), AF488-BrdU (MoBU-1) (1:50; Invitrogen B35110), anti–DNA polymerase η (1:200; Abcam ab236450), and anti-POLD1 (1:200; Abcam ab186407). For RPA2 staining, cells were further incubated with rabbit AF488 secondary antibody (1:250; Invitrogen A-21244) at room temperature for 30 minutes. The stained cells were washed, resuspended in 1× Perm/Wash buffer containing DAPI (1 μg/mL), and acquired on a BD Fortessa flow cytometer.

For ssDNA detection, 5-bromo-2′-deoxyuridine (BrdU; 2 mg) (Invitrogen) was injected into mice i.p. for 16 hours, followed by EdU (1 mg) for the last 2 hours. HSPC staining and pre-extraction were performed as described above. BrdU and EdU dual staining was performed using the BrdU flow kit (BD Biosciences), except DNase I digestion was omitted, followed by EdU staining (Thermo Fisher Scientific C10419) per the manufacturer’s recommendations and analysis by flow cytometry.

### BM transplantation.

For competitive primary BMT, 0.3 × 10^6^ total BM cells (CD45.2) were mixed with an equal number of competitors (CD45.1/CD45.2) and retro-orbitally injected into lethally irradiated recipient mice (CD45.1). Peripheral blood samples were collected at 4-week intervals, and donor reconstitution was analyzed using flow cytometry. After 16 weeks of primary transplantation, recipient mice were sacrificed, and BM cells were enriched for Lin^–^ using the Lineage Cell Depletion Kit (Miltenyi Biotec). Cells were first stained with Live/Dead Fixable Aqua (Invitrogen), followed by surface staining. Intracellular staining was performed to determine intracellular proteins within the donor HSPC populations. For the short-term hematopoietic stress, 15 × 10^6^ total BM cells (CD45.2) were transplanted into lethally irradiated recipient mice (CD45.1). Mice were sacrificed on day 6 and day 10 after BMT and stained for flow cytometry.

### Western blot.

For Western blot, the cell pellets were directly lysed in 1× LDS sample buffer (Invitrogen) with a reducing agent (Invitrogen) and sonicated for homogenization. Samples were boiled at 90°C for 10 minutes, and then the standard Western blot protocol was followed. Membranes were incubated with primary antibodies overnight at 4°C, followed by HRP-conjugated secondary antibodies against rabbit or mouse for 1 hour at room temperature. Membranes were developed with ECL (Thermo Fisher Scientific 34095), and images were captured by KwikQuant Imager. The antibodies used in this study were anti-γH2AX (1:1,000; Millipore 05-636), anti–histone H3 (1:1,000; Cell Signaling Technology 3638S), anti-PCNA (1:1,000; Cell Signaling Technology 13110S), anti-RPA2 (1:1,000; Cell Signaling 2208), anti–pSer4/8 RPA2 (1:1,000; Bethyl A300-245A), anti–pSer33 RPA2 (1:1,000; Bethyl A300-246A), anti–pSer108 MCM2 (1:1,000; Bethyl A300-094A), anti-MCM2 (1:1,000; Bethyl A301-191A), anti–pSer824 KAP1 (1:1,000; Bethyl A300-767A), anti-KAP1 (1:1,000; Bethyl A300-274A), anti-actin (1:2,000; Santa Cruz Biotechnology sc-8432), anti-p53 (1:1,000; Cell Signaling Technology 2524S), anti–pSer345 CHK1 (1:1,000; Invitrogen MA5-15145), anti-CHK1 (1:1,000; Cell Signaling Technology 2360S), and anti–DNA polymerase η (1:200; Abcam ab236450).

### Viral transduction of LSK cells and BMT.

Sorted LSK cells from WT and *Lnk^–/–^* mice were cultured for 2 days in SFEM medium (Stemcell Technologies Inc.) supplemented with 10% FBS (SAFC Biosciences) and cytokines (100 ng/mL mSCF, 20 ng/mL mTpo, 20 ng/mL FLT3L, 20 ng/mL IL-6). Lentivirus expressing microRNA-30–based shRNA to luciferase (Luc) or *PolH* with mCherry as a marker was preloaded twice into a 12-well plate coated with RetroNectin (TaKaRa T100B) (62). The shRNA sequences were: sh*Polh*#1, CGCATTTGGTGTCACTAGAAAC, and sh*Polh*#2, CCCAGATCTTCTCCTGGCACAA. Cultured LSK cells were transferred to the lentivirus-preloaded plates and incubated for 1 more day. At day 3, 0.3 × 10^6^ cultured LSK cells (CD45.2) were mixed with 0.6 × 10^6^ Sca1-depleted competitor BM cells (CD45.1/CD45.2) and injected into recipient mice (CD45.1) that were irradiated with a split dose of 10 Gy. A small fraction of infected cells was kept for 1 more day to evaluate the viral infection efficiency (56). After BMT, peripheral blood samples were collected every 4 weeks, and the reconstitution of mCherry^+^ cells within total peripheral blood and mCherry^+^ percentages within CD45^+^ donors were analyzed using LSR Fortessa flow cytometry.

### HSC sorting and transplantation.

HSC purification and BMT were performed as described previously (14). Briefly, Lin^+^ cells were first depleted using a Lineage Cell Depletion Kit (Miltenyi Biotec catalog 130-090-858). Lin^–^ cells were then stained with APC–Cy7–c-Kit (2B8), PerCP-Cy5.5-Sca1 (E13-161.7 or D7), FITC-CD48 (HM48-1), and PE-Cy7-CD150 (TC15-12F12.2). One hundred fifty HSCs (CD150^+^CD48^–^LSK) were sorted into a round-bottom 96-well plate on a FACSAria Fusion sorter (BD Biosciences). HSCs were cultured for 3 days in 5 μM REV1i or DMSO as a control. Cells from each well were mixed with 0.6 × 10^6^ Sca1-depleted competitor BM cells (CD45.1/2) and injected retro-orbitally into lethally irradiated recipient mice (CD45.1). Peripheral blood samples were collected every 4 weeks, and donor reconstitution was analyzed on a FACS Fortessa or Cytek Aurora flow cytometer.

### Colony assays.

Mouse BMs were plated in semi-solid methylcellulose culture (Stemcell Technologies Inc.) according to the manufacturer’s recommendations with M3434 medium. The colony numbers were scored 7–12 days later.

### pIpC injection.

For repeated pIpC injection, 5 mg/kg pIpC (InvivoGen) was administered i.p. The administration regimen was 2 times per week for 4 weeks, followed by 4 weeks of rest, and this 8-week cycle was repeated 7 times. For a single pIpC injection to induce HSC into the cell cycle, 2 mg/kg was administered i.p.

### Fork restart flow cytometry assay.

Sorted LSK cells were cultured for 3 days in SFEM medium supplemented with 10% FBS together with various cytokines: mSCF (100 ng/mL), mTPO (20 ng/mL), mIL-6 (20 ng/mL), and mIL-3 (20 ng/mL) (PeproTech Inc.). After 3 days, cells were pulsed with 10 μM BrdU (Invitrogen) for 10 minutes to label the S-phase cells. Cells were washed once with IMDM containing 10% FCS and incubated with hydroxyurea (HU; 0.5 mM) (MilliporeSigma) for 1 hour to halt the S-phase progression. Cells were washed twice and further incubated with EdU (10 μM) (Click Chemistry Tool) for 1 hour to assess the ability to restart the replication within S-phase cells (BrdU^+^). Cells were immediately fixed and stained with cell surface PerCP-Cy5.5-Sca1 (D7) and APC-Cy7-CD48 (HM48-1) antibodies. BrdU and EdU dual staining was performed using the BrdU flow kit (BD Biosciences) with anti-BrdU (Invitrogen MoBU-1) antibody, followed by EdU staining (Thermo Fisher Scientific C10419) per the manufacturer’s recommendations, and analyzed by flow cytometry.

### Single-molecule DNA fiber assay.

Fork recovery and ssDNA gap assays of single-molecule DNA fibers were performed similarly to published protocols (63, 64). Briefly, freshly isolated LSK cells were recovered in cytokine-containing medium for 1 hour before fiber assay. For the fork recovery assay, replicating DNA was first labeled by a 20-minute pulse with 50 μM CldU (MilliporeSigma), followed by the addition of 2 mM HU (MilliporeSigma) for 1 hour to stall replication. Cells were then washed and pulsed with 250 μM IdU (MilliporeSigma) for 20 minutes. For S1 nuclease assay, replicating DNA was first labeled by a 20-minute pulse with 50 μM CldU, followed by a 40-minute pulse with 250 μM CldU in the presence of 20 μM HU. Cells were then collected, washed with CSK buffer (100 mM NaCl, 10 mM MOPS, 3 mM MgCl_2_ pH 7.2, 300 mM sucrose, 0.25% Triton X-100), and subjected to a 30-minute treatment with or without S1 nuclease. For both assays, cells were lysed directly on silane-coated slides and then tipped to 30° to spread single DNA fibers. Slides were subsequently fixed, and DNA denatured and neutralized. Slides were incubated with anti-CldU (rat, Abcam) and IdU (mouse, BD Biosciences) primary antibodies, followed by goat anti-rat AF488 and goat anti-mouse AF568 (Invitrogen) secondary antibodies. DNA fibers were captured using a ×100 or ×60 objective on a Nikon Eclipse 80i fluorescent microscope and quantified using ImageJ software (Fiji).

### Immunofluorescence.

Ex vivo–cultured HSPCs were fixed with 4% paraformaldehyde for 20 minutes at 4°C and washed with PBS. Fixed cells were permeabilized with 0.2% Triton X-100 for 5 minutes on ice. Cells were washed and stained with anti–mouse γH2AX antibody (1:200; Millipore 05-636) for 2 hours at room temperature, followed by 3 washes with PBS. Cells were stained with anti-mouse Cy3 secondary antibody (715-165-150, Jackson ImmunoResearch) for 30 minutes at room temperature. Cells were washed twice, and nuclei were stained with DAPI. Stained cells were spun onto glass microscope slides and visualized using fluorescent microscopes. All the captured images were analyzed using Fiji software.

### Statistics.

For all cell culture, colony-forming cell assays, and BMT assays, 2-tailed Student’s *t* tests or ANOVA with multiple comparisons were performed. Graphs are presented as mean ± SEM or mean ± SD. For DNA fiber ratios in the DNA fiber assays, the Kruskal-Wallis 1-way ANOVA test was used for non-parametric data, and comparisons between individual groups were calculated using Dunn’s multiple-comparison post-test in Prism (GraphPad Software Inc.). Statistical significance was determined by 2-tailed Student’s *t* test, and a *P* value less than 0.05 was considered statistically significant.

### Study approval.

All the animal studies were performed under a protocol approved by the Institutional Animal Care and Use Committee of Children’s Hospital of Philadelphia (2024-0781).

### Data availability.

Please refer to the Supporting Data Values file containing all of the underlying values for the data presented in the article. There are no large datasets or computer codes associated with this work.

## Author contributions

WT conceived the project and supervised the studies. WT, BS, MAH, and RAG designed the experiments and wrote the manuscript with input from all authors. BS and MAH performed all the animal experiments with the assistance of CSS and XHL, who did animal husbandry. BS performed biochemistry, and JF performed colony assays and DNA fiber assays. XHL performed BMT and peripheral blood sampling.

## Funding support

This work is the result of NIH funding, in whole or in part, and is subject to the NIH Public Access Policy. Through acceptance of this federal funding, the NIH has been given a right to make the work publicly available in PubMed Central.

- NIH grants R01DK127738 and R01HL095675 to WT and R01 CA174904 to RAG.
- Awards from the Basser Center for BRCA Research to WT.
- Distinguished chair in pediatric hematology of Children’s Hospital of Philadelphia to WT.
- Hematopoiesis training grant (NIH T32DK007780) to MAH.
- NIH TL1DK143326 and U2CDK136784 to MAH.

## Supplementary Material

Supplemental data

Unedited blot and gel images

Supporting data values

## Version 1 10/30/2025

In-Press Preview

## Version 2 01/02/2026

Electronic publication
