# Supplementary material for: Lnk deficiency enhances translesion synthesis to alleviate replication stress and promote hematopoietic stem cell fitness
Source: J Clin Invest. 2025 Oct 30;136(1):e191713. doi: 10.1172/JCI191713 (PMC12721908; doi:10.1172/JCI191713)
Supplement: Supplemental data [file jci-136-191713-s238.pdf]

Supplementary figures for

Lnk deficiency Enhances Translesion Synthesis to alleviate Replication Stress and Promote Hematopoietic Stem Cell Fitness

Brijendra Singh [1,2,#], Md Akram Hossain [1,2,#], Xiao Hua Liang [1,2], Jeremie Fages [1,2], Carlo Salas Salinas[1,2], Roger A. Greenberg[3], and Wei Tong [1,2,4,*]

[1] Division of Hematology, Children's Hospital of Philadelphia, Philadelphia, PA 19104

[2] Division of Pediatrics, Perelman School of Medicine at the University of Pennsylvania, Philadelphia, PA 19104

[3] Division of Cancer Biology, Perelman School of Medicine at the University of Pennsylvania, Philadelphia, PA 19104

[4] Lead Contact

[*] Correspondence author: tongw@chop.edu

Keywords

Hematopoietic stem cells (HSCs), replication stress, translesion synthesis, DNA replication, DNA damage response, DNA repair, DNA damage tolerance, stem cell fitness.

Running title: LNK regulates replication stress in HSCs

Figure S1, related to figure 3.

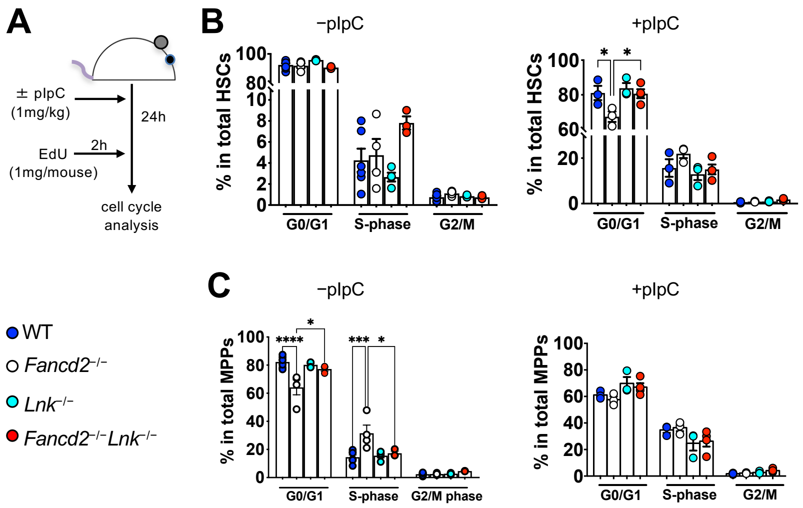

Figure S1. Cell cycle analysis of HSCs and MPPs with and without pIpC administration. (A) Experimental design for measuring the activation of the ATR/ATM pathway in different cell cycle stages upon pIpC-induced HSPC replication, as in Figure 2. **(B-C)** Quantification of cell cycle stages in the HSCs **(B)** and MPPs **(C)** of different genotypes of mice without and with pIpC-induced replication stress. In all relevant panels, each symbol represents an individual mouse; bars indicate mean values. Each symbol represents an individual mouse. Bars indicate mean values, and error bars indicate SEM. p values were calculated using one-way ANOVA, *, p<0.05; **, p<0.01.

Figure S2, related to Figure 3.

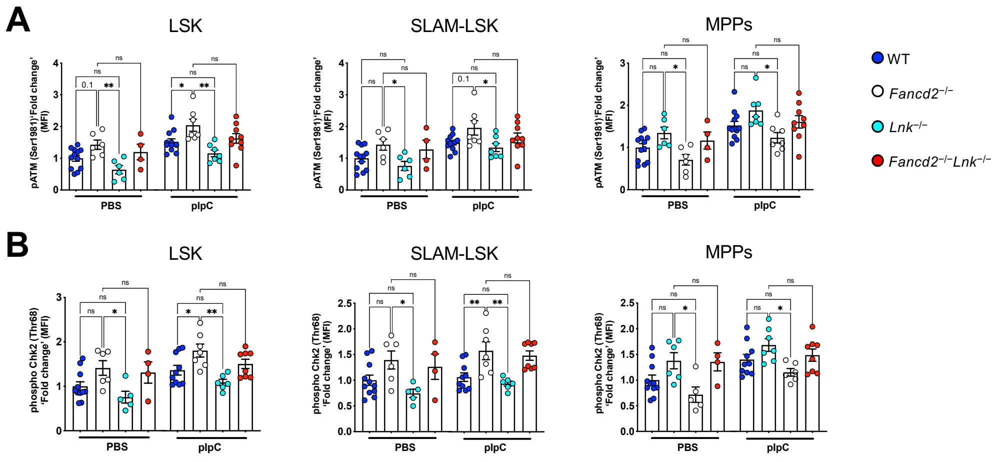

Figure S2. Loss of *Lnk* does not reduce the activation of the ATM pathway in *Fancd2*$^{-/-}$ HSPCs upon pIpC-induced replication stress. Quantification of pATM (Ser1981) **(A)** and pCHK2 (Thr68) **(B)** (fold change in MFI) within LSK, SLAM-LSK, and MPPs populations of PBS and pIpC administrated mice. Pooled data from three independent experiments are expressed as mean ± SEM. Each symbol represents an individual mouse. Bars indicate mean values, and error bars indicate SEM. p values were calculated using one-way ANOVA, *, $p<0.05$; **, $p<0.01$; ***, $p<0.001$.

Figure S3, related to Figure 4.

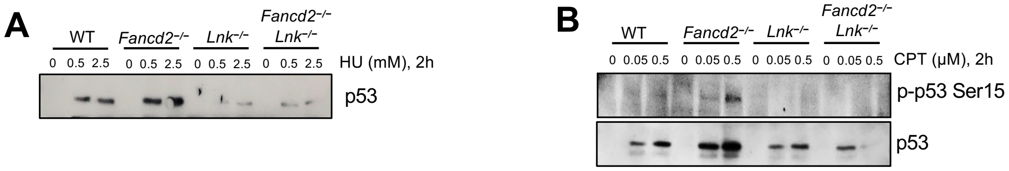

Figure S3. *Lnk* deficiency reduces p53 activation in *Fancd2*$^{-/-}$ HSPCs. Representative immunoblots showing the phosphor-p53 (p-p53) and total p53 levels in freshly sorted LK cells treated with increasing concentrations of HU **(A)** or CPT **(B)**. The images in **A** and **B** were derived from the same experiment initially shown in Fig. 1H left and right panels, respectively.

Figure S4, related to Figure 5.

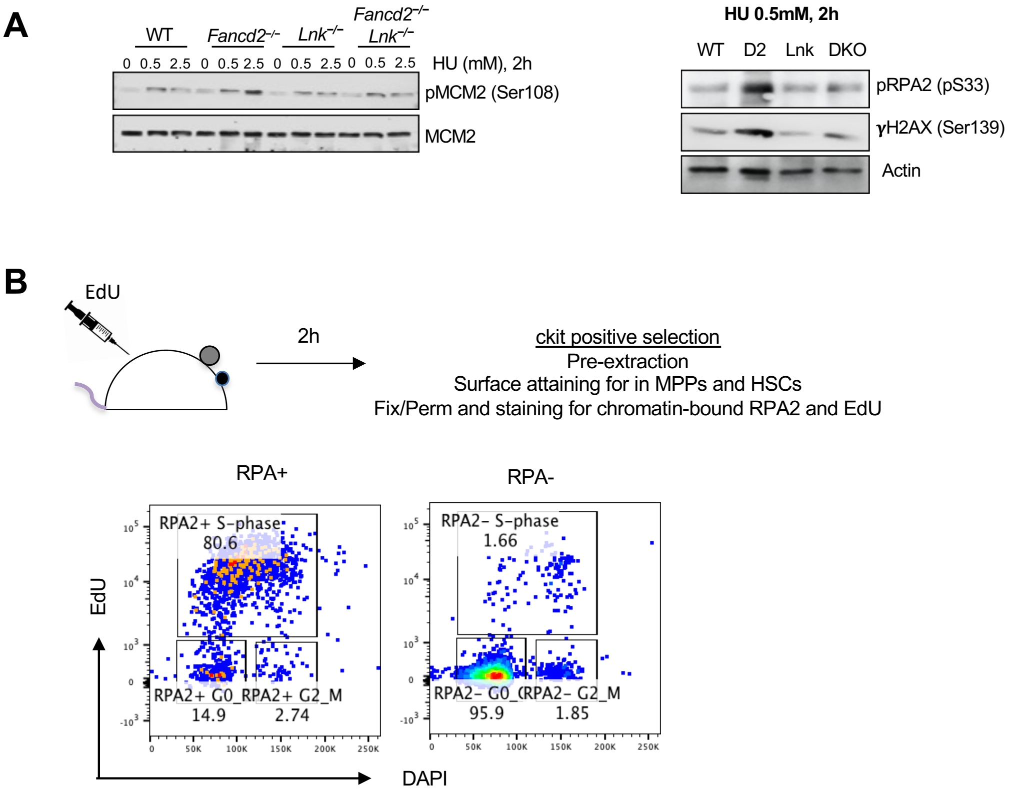

Figure S4. *Lnk* deficiency reduces replication stress and RPA accumulates on the chromatin in the S phase. (A) Immunoblots of whole cell lysates showing the expression of pMCM2 (Ser108) and pRPA(S33) in freshly sorted LK cells treated with a graded concentration of HU, cultured in the presence of cytokines (SCF, TPO, IL-3, IL-6) for 2h. The images were derived from the same experiment initially shown in Fig. 3G (left panel). **(B)** Experimental scheme to measure chromatin-bound RPA in different cell cycle phases in HSPCs in vivo. Representative flow cytometry plot showing the cell cycle of RPA+ HSCs (left) and RPA- HSCs (Right).

Figure S5, related to Figure 6.

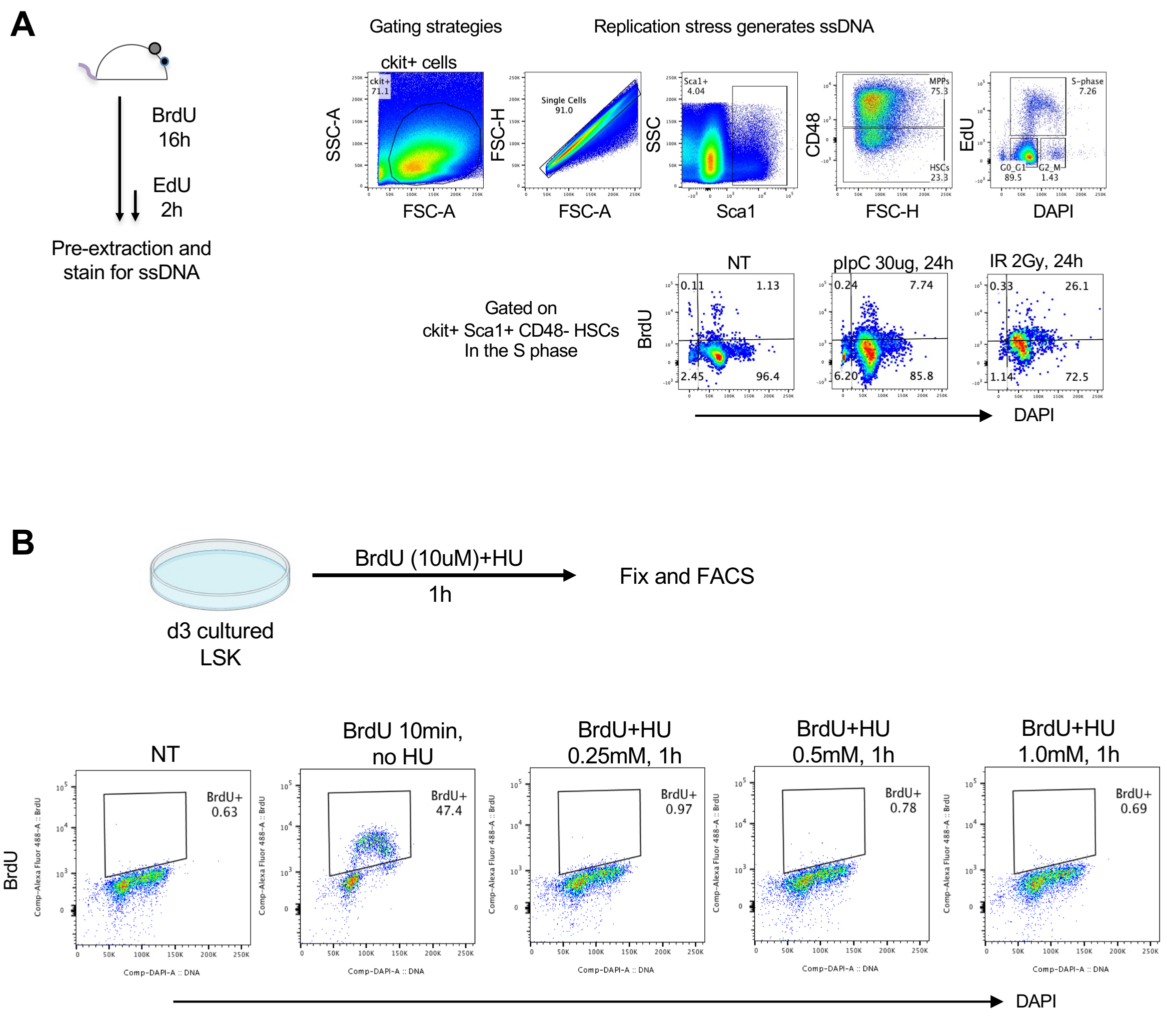

Figure S5. A flow cytometry assay to measure ssDNA in HSPCs and titration of HU to stall replication in primary HSPCs. (A) Schematic outline of the experimental procedure for measuring replication-stress (pIpC or IR) induced ssDNA within the HSPC population (Left). Gating strategy and representative flow cytometry plots to examine ssDNA in the S phase of HSCs (Right). The S-phase cells are gated for the EdU+ population, and neutralized BrdU staining indicates ssDNA. NT: no treatment. **(B)** Schematic outline of the experimental procedure to titrate HU doses to stall replication for the fork recovery flow cytometry assay. Flow plots show the BrdU incorporation in cultured Kit$^+$Sca1$^+$ HSPCs in the absence or presence of a graded dose of HU.

Figure S6, related to Figure 8.

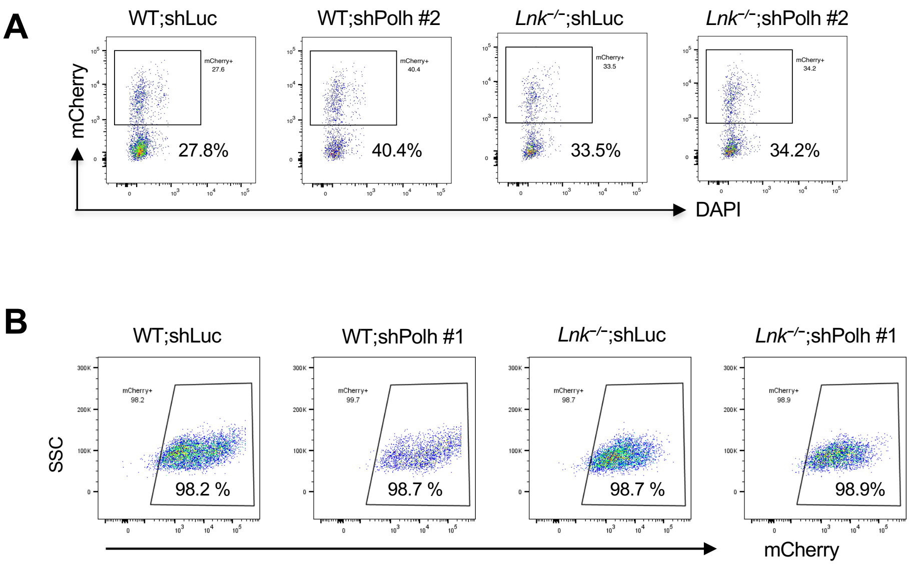

Figure S6. Infection rates of shRNA-mediated *PolH* knockdown in HSPCs. Flow cytometric plots showing the infection rates at the time of transplantation for two different BMTs using different shRNAs to PolH, #2 **(A)** and #1 **(B)**.

Figure S7.

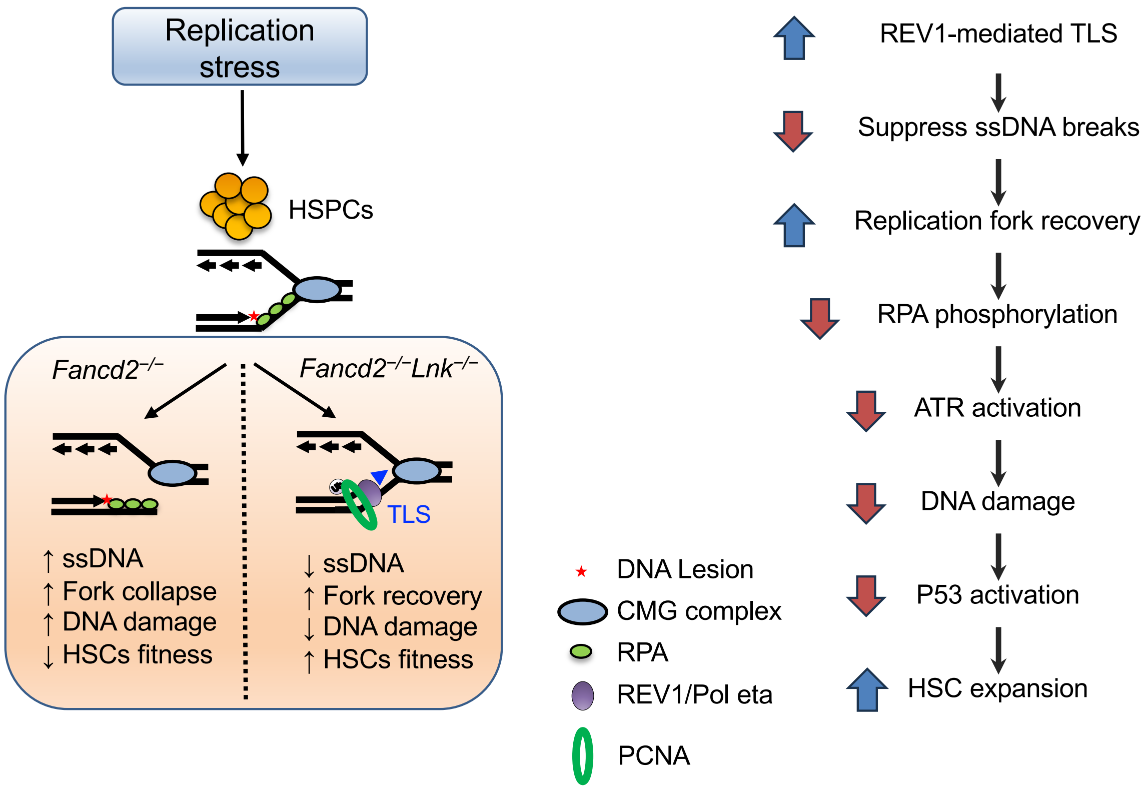

Figure S7. Working model to show *Lnk* deficiency reduces replication stress and promotes HSC fitness via enhancing REV1-mediated TLS.
