# Supplementary material for: Lnk deficiency enhances translesion synthesis to alleviate replication stress and promote hematopoietic stem cell fitness
Source: J Clin Invest. 2025 Oct 30;136(1):e191713. doi: 10.1172/JCI191713 (PMC12721908; doi:10.1172/JCI191713)

Figure 1H. Singh, et al

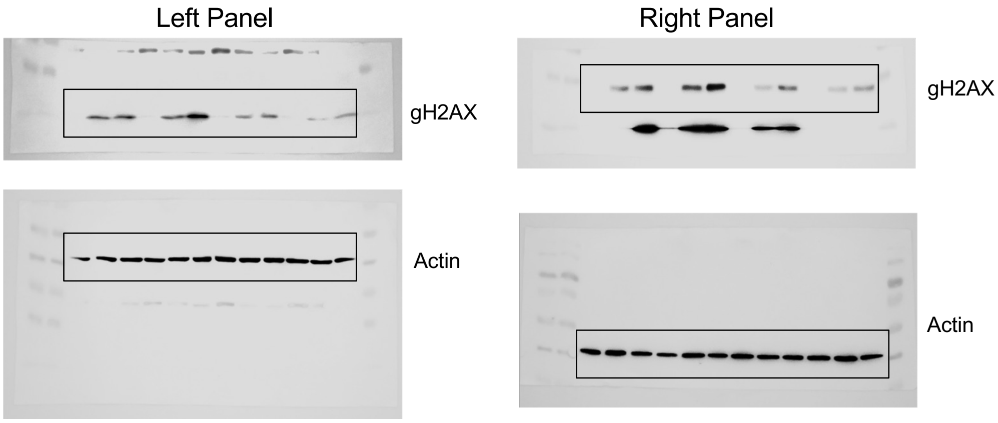

Figure 3. Singh, et al

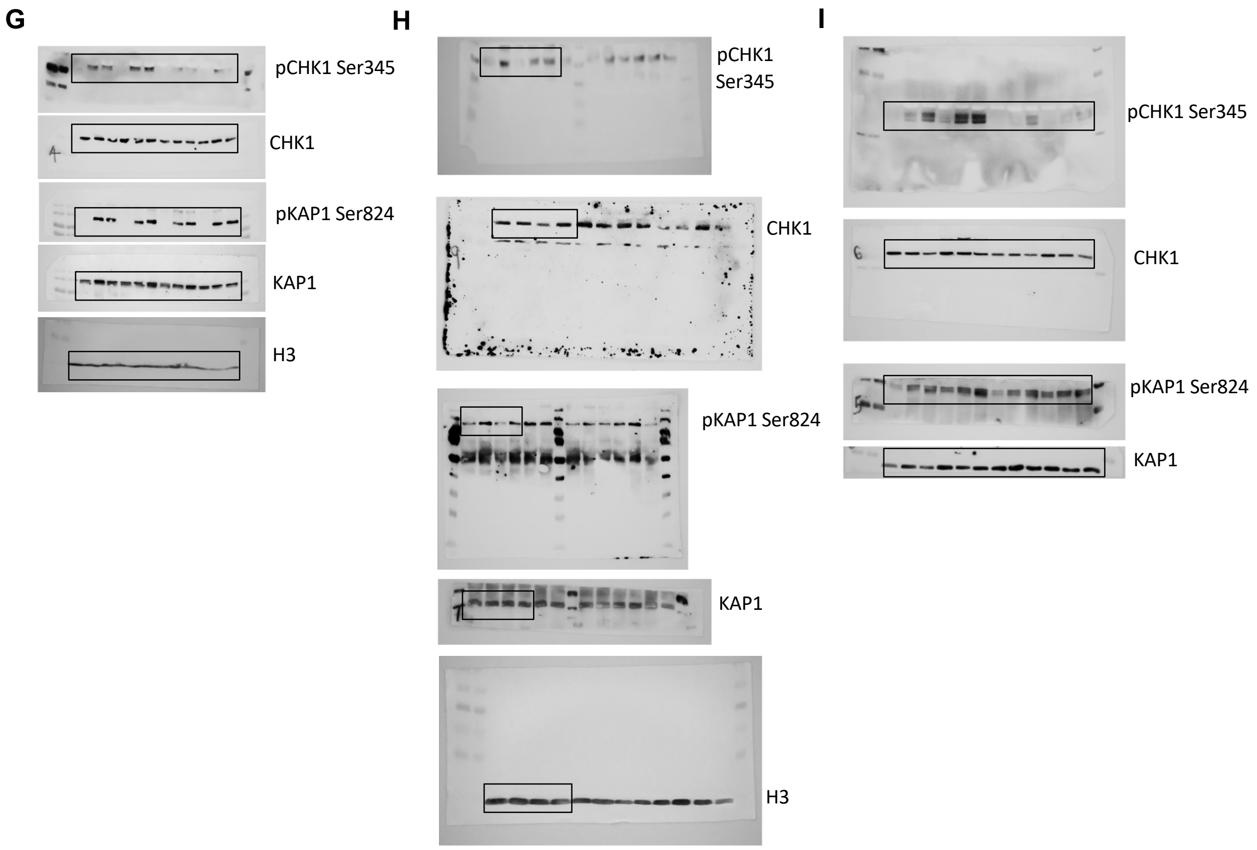

Figure 5. Singh, et al

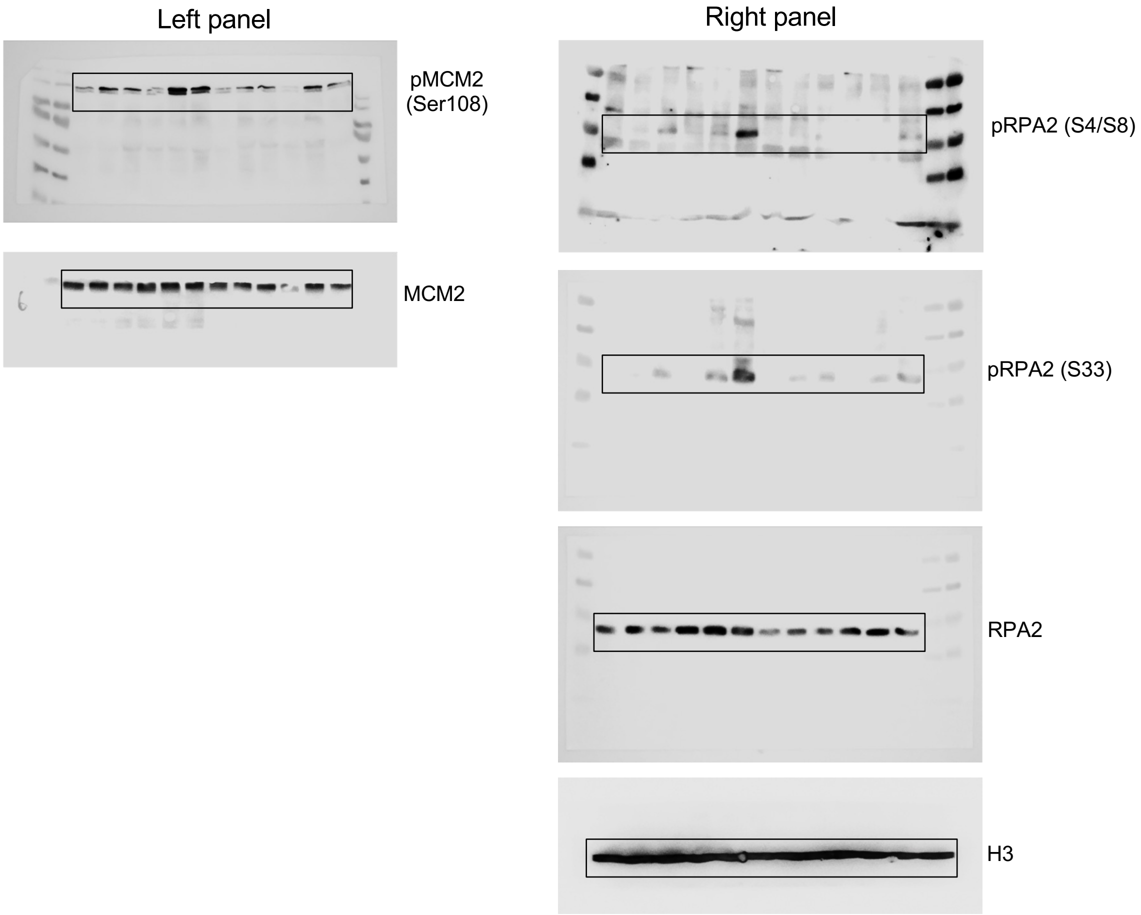

Figure 8C. Singh, et al

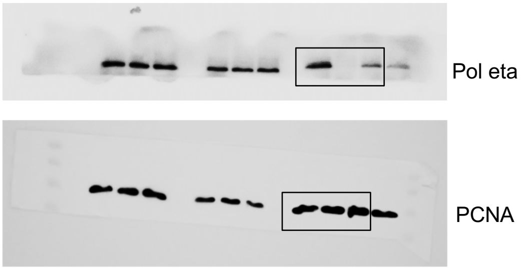

Figure S3, related to Figure 4.

A

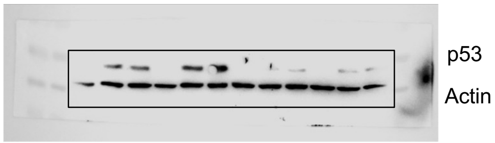

B

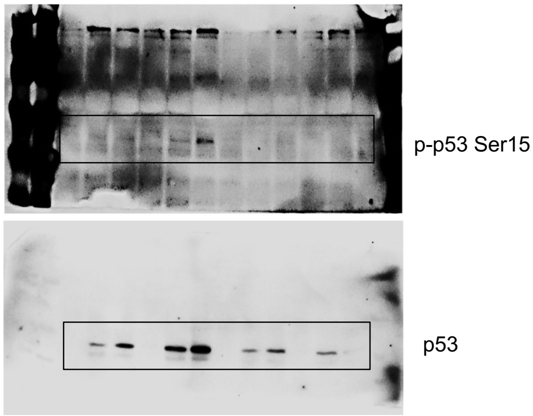

Figure S4, related to Figure 5.

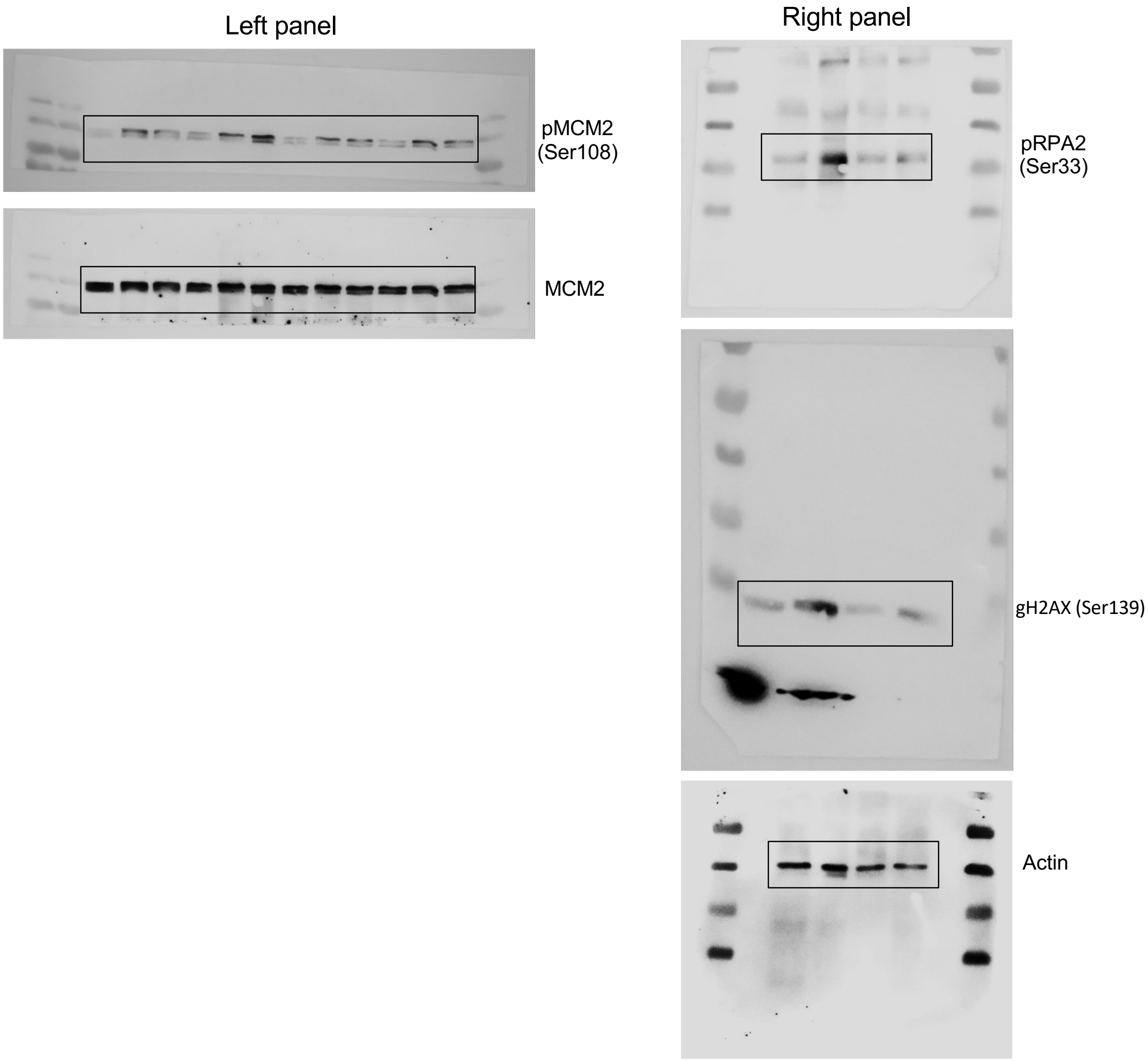

Supplement: Unedited blot and gel images [file jci-136-191713-s239.pdf]
